# 90K, an interferon-stimulated gene product, reduces the infectivity of HIV-1

**DOI:** 10.1186/1742-4690-10-111

**Published:** 2013-10-24

**Authors:** Veronika Lodermeyer, Kristina Suhr, Nicola Schrott, Christian Kolbe, Christina M Stürzel, Daniela Krnavek, Jan Münch, Christian Dietz, Tanja Waldmann, Frank Kirchhoff, Christine Goffinet

**Affiliations:** 1 Institute of Molecular Virology, Ulm University Medical Center, Ulm, Germany; 2 Nycomed Chair of Bioinformatics and Information Mining, University of Konstanz, Konstanz, Germany; 3 Doerenkamp-Zbinden Department of in vitro Toxicology and Biomedicine, University of Konstanz, Konstanz, Germany; 4 TWINCORE, Institute of Experimental Virology, Feodor-Lynen-Strasse 7, 30625 Hannover, Germany

**Keywords:** Interferon, HIV-1, Antiviral factor, Env incorporation

## Abstract

**Background:**

In response to viral infections, interferons induce the transcription of several hundred genes in mammalian cells. Specific antiviral functions, however, have only been attributed to a few of them. *90K/LGALS3BP* has been reported to be an interferon-stimulated gene that is upregulated in individuals with cancer or HIV-1 infection.

**Results:**

Here, we show that 90K expression dose-dependently decreased the particle infectivity of HIV-1 progeny. The lower infectivity of released particles correlated with reduced virion incorporation of mature envelope glycoproteins gp120 and gp41. Further, proteolytic processing of the gp160 precursor and surface expression of gp120 in the producer cell were impaired in the presence of 90K expression. In contrast, expression of Gag, Nef and Vpu, and virus release were not grossly affected by 90K expression. 90K-imposed restriction occurred in the absence of direct interaction of 90K with HIV-1 Env or entrapment of Env in the ER. The cell-associated, but not the secreted species of 90K, mediated the antiviral effect. A truncated version of human 90K, solely consisting of the two intermediate domains, displayed a similar antiviral activity as the full-length wildtype 90K, indicating that the N-terminal SRCR-like domain and the C-terminal domain are dispensable for 90K’s antiviral activity. The murine homolog of 90K, CypCAP (Cyclophilin C-associated protein), neither modulated particle infectivity of HIV-1 nor lowered the virion incorporation of mature gp120, suggesting a species-specific mode of action. 90K was expressed at basal levels in TZM-bl cells and in primary macrophages, and at low levels in CD4^+^ T-cells and PBMCs. 90K’s susceptibility to IFN-mediated stimulation of expression was cell type-specific. siRNA-mediated knockdown of 90K in TZM-bl cells and primary macrophages enhanced the incorporation of Env glycoproteins into progeny virions, boosted the particle infectivity of released HIV-1, and accelerated HIV-1 spread. Conversely, treatment of HIV-1 infected macrophages with IFN-α induced 90K expression and lowered the particle infectivity of HIV-1.

**Conclusions:**

Thus, 90K constitutes a novel antiviral factor that reduces the particle infectivity of HIV-1, involving interference with the maturation and incorporation of HIV-1 Env molecules into virions.

## Background

Human cells are equipped with antiviral proteins that are capable of disturbing various steps of the replication cycle of diverse viruses. Notable examples of anti-HIV-1 factors include members of the apolipoprotein editing complex (APOBEC) class of cytidine deaminases, which induce lethal hyper-mutations of the viral genome [1], Tetherin/CD317/BST2, which causes retention of viral particles on the infected cell’s surface [2,3], and SAMHD1, that prevents reverse transcription in myeloid cells and resting CD4^+^ T-cells [4-6]. Expression of these restriction factors is typically interferon (IFN)-responsive [7], and HIV-1 infection results in their enhanced transcription both *ex vivo*[8] and *in vivo*[9].

*90K/LGALS3BP,* which encodes for a secretory glycoprotein of 585 amino acids, shares these properties. By virtue of an IFN-responsive element in the *90K* promoter [10], levels of mRNA are enhanced by type I and type II IFNs in PBMCs [11-13]. Induction of 90K protein expression by IFN-α has been shown in carcinoma cell lines [14,15] as well as *in vivo* following IFN-α administration to cancer patients [14,16]. Furthermore, HIV-1 infection has been reported to potentiate expression of 90K in primary CD4^+^ T-cell cultures [8] as well as *in vivo* in HIV-1 infected individuals [9,17-22], although it remains unclear whether the enhanced 90K levels can be attributed to HIV-1-infected cells or uninfected bystander cells *in vivo*.

90K, originally identified as a tumor-associated antigen [14,23,24], is a member of the family of scavenger receptor cysteine-rich (SRCR) domain-containing proteins [25]. It enters the secretory pathway by virtue of its N-terminal signal peptide and is *N*-glycosylated in the endoplasmic reticulum and Golgi complex [26]. Secreted 90K protein is present in the μg/ml range in several body fluids of healthy individuals, including blood, semen, urine, tears, saliva, and breast milk [18,27,28]. Its physiological functions seem to comprise roles in immune response modulation and cell adhesion. Specifically, secreted 90K promoted NK cell activity, CD8^+^ T-cell-mediated lysis activity and cytokine production [11]. Furthermore, secreted 90K induced homotypic cell adhesion and the formation of multicellular aggregates of tumor cells [29] by binding to galectin-3/Mac-2.

The inducibility of 90K expression by IFNs and its upregulated expression in HIV-1 infected individuals prompted us to investigate whether 90K modulates HIV-1 replication. In this study, we establish 90K as an antiviral factor that is associated with the release of poorly infectious virions. This involved defective maturation of HIV-1 Env during *de novo* virus production and reduced incorporation of Env into progeny virions. The two central protein-binding domains of 90K were essential and sufficient for its antiviral infectivity. 90K protein expression was cell type-specific, and the degree of IFN-mediated upregulation of *90K* at the protein level differed between macrophages and CD4^+^ T-cells. Depletion of endogenous 90K in HIV-1 susceptible cells, including TZM-bl cells and primary macrophages, boosted the particle infectivity of progeny HIV-1, ameliorated Env incorporation into nascent virions and accelerated the spread of HIV-1. Reduction of 90K expression in PBMCs likewise enhanced HIV-1 spread. Concordantly, IFN-α treatment of primary HIV-1 infected macrophages lowered the particle infectivity of viral progeny. These data suggest that 90K is an important contributor of the cellular antiviral defense against HIV-1 infection.

## Results

### 90K expression reduces the particle infectivity of HIV-1 progeny by interfering with the maturation and incorporation of the viral Env glycoproteins into progeny virions

**Figure 1:**
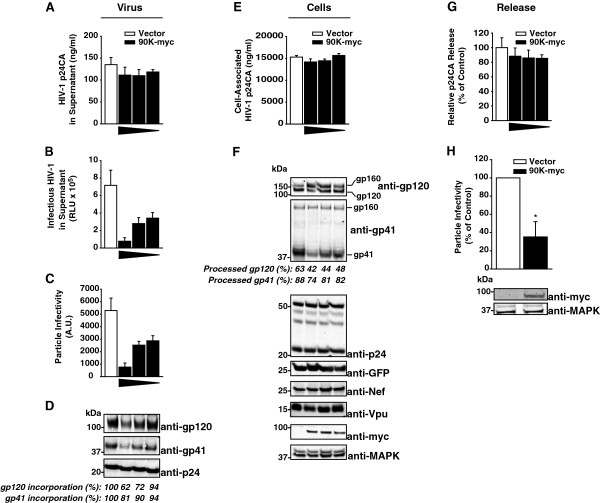
**Heterologous expression of 90K reduces the particle infectivity of HIV-1 progeny by interfering with the maturation and incorporation of the viral glycoproteins gp120 and gp41. (A)** 293T cells were cotransfected with 1.3 μg pBR.HIV-1 IRES GFP and various amounts of pcDNA6.90K-myc (1.3, 0.4, 0.15 μg) or empty vector. The total amount of DNA was kept constant (2.6 μg). Supernatants were analyzed for amounts of released HIV-1 capsid antigen by anti-p24 capsid ELISA and **(B)** infectious HIV-1 by applying a TZM-bl luciferase assay. **(C)** Particle infectivity was defined as infectivity per ng released capsid. Shown are arithmetic means ± S.D. of triplicates. **(D)** Sucrose cushion-purified virions were analyzed by immunoblotting. Percentages indicate the relative gp120 and gp41 incorporation, as measured by Infrared imaging-based quantification of the amount of gp120 and gp41 per p24, respectively. The values of incorporation in absence of 90K expression were set to 100%. **(E)** Corresponding cell lysates were analyzed for cell-associated p24 capsid by ELISA. **(F)** Cell lysates were analyzed by immunoblotting using the indicated antibodies. Numbers indicate the efficiency of gp120 and gp41 processing, respectively, calculated as the signal ratio of gp120 relative to (gp120 + gp160), or of gp41 relative to (gp41 + gp160), respectively. **(G)** Capsid release was calculated as p24 capsid antigen in the supernatant (p24_Sup_) to total p24 capsid (p24_Cell_ + p24_Sup_). The condition without 90K was set to 100%. **(H)** SupT1 cells were electroporated with pBR.HIV-1 IRES GFP (10 μg) and pcDNA6.90K-myc or empty vector (10 μg). Three days post transfection, supernatants were harvested and analyzed for particle infectivity by applying a TZM-bl infectivity assay and an anti-p24 capsid ELISA of the sucrose cushion-purified supernatants. Western Blot analysis of the producer cells was performed using the indicated antibodies.

In order to investigate whether 90K may affect HIV-1 replication, 293T cells were cotransfected with a proviral HIV-1 DNA and decreasing amounts of an expression plasmid for C-terminally myc-tagged 90K (90K-myc). Notably, transfection of 293T cells with a 90K-encoding plasmid resulted in levels of 90K expression that were clearly higher than those obtained following IFN stimulation (Additional file 1: Figure S1A). Anti-p24 capsid ELISA analysis showed that 90K-myc expression did not modulate the levels of released HIV-1 capsid in the supernatant (Figure 1A). In contrast, 90K-myc decreased the quantity of infectious HIV-1 released in the culture supernatant (Figure 1B). Consequently, the particle infectivity (infectivity per ng p24CA) was dose-dependently reduced by 90K-myc expression (Figure 1C).

Anti-HIV-1 p24 capsid Western Blotting of sucrose cushion-purified supernatants of the producer cells confirmed that 90K-myc did not affect levels of released viral p24 capsid antigen but, in contrast, induced a dose-dependent decrease in virus-associated gp120 and gp41 Env proteins (Figure 1D).

ELISA analysis of corresponding cell lysates did not show any detectable effect of 90K expression on cell-associated levels of HIV-1 capsid (Figure 1E). Calculation of HIV-1 capsid release showed that 90K did not interfere with HIV-1 release (Figure 1G). However, a profound impact of 90K expression on the relative proportion of the uncleaved gp160 precursor and its cleavage products was disclosed (Figure 1F). Specifically, expression of 90K-myc reduced the relative amounts of mature gp120 and gp41, respectively, whereas it increased the relative levels of uncleaved gp160 precursor (Figure 1F). Of note, the effect on gp41 processing was generally lower than on gp120, possibly due to increased sensitivity of the anti-gp41 antibody to cleaved gp41. Thus, 90K expression affected the proteolytic maturation and virion incorporation of the HIV-1 Env glycoproteins. Importantly, 90K specifically inhibited Env biosynthesis, since neither the expression levels of other viral proteins, including HIV-1 Nef, Gag and Vpu, nor Gag processing were grossly affected by 90K-myc expression (Figure 1F). Further, expression of the *gfp* reporter gene was not affected by 90K-myc, demonstrating that global cellular protein biosynthesis was not disturbed. 90K-myc expression also reduced the particle infectivity of R5-tropic HIV-1_LAI_ and HIV-1_YU-2_, as well of three transmitted/founder HIV strains (Additional file 2: Figure S2), indicating that its antiviral activity is not restricted to viruses of a specific tropism or to laboratory-adapted viral strains.

90K-myc-mediated inhibition of Env biosynthesis and of Env virion incorporation occurred in the absence of any detectable signs of cytoxicity, as assessed by 7-AAD stain and a luminometric metabolism assay of 90K-myc-expressing cells (Additional file 3: Figure S3). We next tested whether expression of 90K-myc in a HIV-1-susceptible T-cell line might equally result in a reduction of HIV-1 infectivity. For this purpose, we cotransfected the T-cell line SupT1 with HIV-1 proviral DNA and an expression vector for 90K-myc or the respective empty vector control. Sucrose cushion-purified virions from 90K-myc-expressing producer cells displayed reduced particle HIV-1 infectivity, compared to empty vector-expressing cells (Figure 1H). Thus, 90K is also able to reduce the specific infectivity of progeny HIV-1 in T-cells.

**Figure 2:**
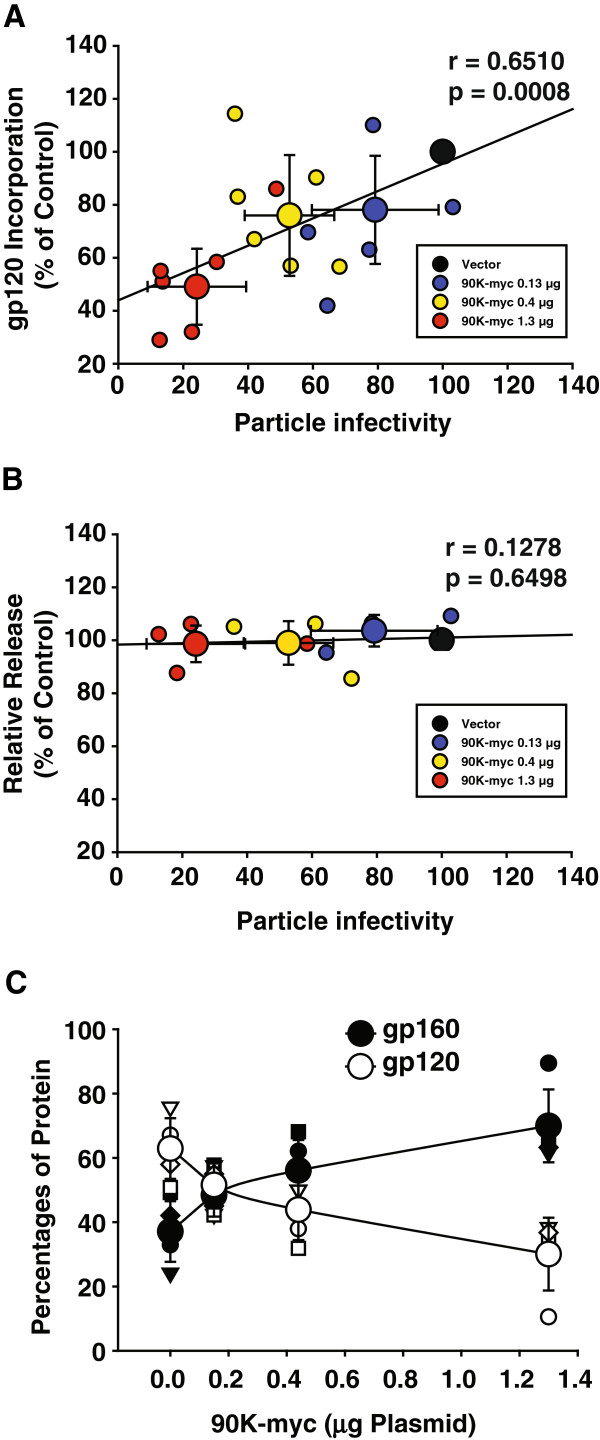
**90K-induced reduction of HIV-1 particle infectivity correlates with defective gp120 incorporation into progeny, without influencing virus release. (A)** Correlative analysis of gp120Env content per p24CA and HIV-1 particle infectivity (infectivity per ng p24) in the supernatant as a function of 90K plasmid amount. Small symbols represent the values of each individual transfection; large symbols represent the arithmetic means ± S.E.M. of five-six individual experiments. The Pearson’s correlation coefficient r and the corresponding p value were calculated using GraphPad Prism Software. **(B)** Correlative analysis of relative released virus and HIV-1 particle infectivity (infectivity per ng p24) in the supernatant as a function of 90K plasmid amount. Small symbols represent the values of each individual transfection; large symbols represent the arithmetic means ± S.E.M. of four individual experiments. The Pearson’s correlation coefficient r and the corresponding p value were calculated using GraphPad Prism Software. **(C)** Relative percentage of cell-associated gp120Env or gp160 Env versus total detected anti-gp120 signal as a function of cotransfected 90K plasmid amount. Values were obtained by Infrared imaging-based quantification of signals. Small symbols represent the values of each individual experiment; large symbols represent the arithmetic means ± S.E.M. of four individual experiments.

Infrared imaging-based protein quantification of Western Blots from multiple experiments corroborated that 90K-myc expression dose-dependently suppressed both the particle infectivity of released HIV-1, and the incorporation of mature gp120 into virions. A statistical analysis established a significant correlation of both parameters in a 90K-dose dependent manner (Figure 2A). Importantly, HIV-1 release was not affected (Figure 2B). Quantification of the signal intensities for cell-associated gp120 and gp160 confirmed that 90K dose-dependently reduced the proportion of processed, mature gp120 relative to the uncleaved gp160 precursor from 63% to 30% (Figure 2C).

**Figure 3:**
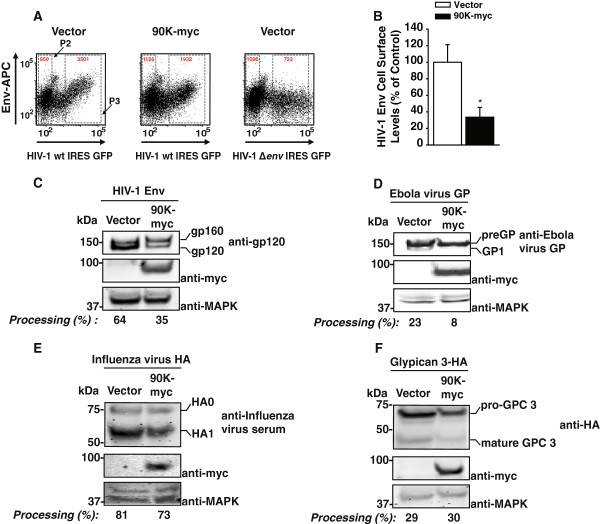
**90K-myc reduces cell surface levels of HIV-1 Env and is not a global inhibitor of furin-mediated proteolytic activity. (A)** 293T cells were cotransfected with pBR.HIV-1 IRES GFP and vector or pcDNA6.90K-myc. Two days post transfection, levels of surface HIV-1 gp120 were assessed by flow cytometry following staining with a human anti-HIV-1 Env antibody. Shown are representative dot plots from one experiment out of three. Red numbers indicate the MFI in gate P2 (HIV-negative cells) and P3 (HIV-positive cells). **(B)** Quantitative analysis of relative HIV-1 Env surface levels in the absence or presence of 90K-myc. Env cell surface levels were calculated by comparing, within the same sample, Env levels on non-GFP-expressing cells (gate P2) with Env levels on cells with medium-high expression levels (gate P3). The ratio obtained for vector-transfected cells was set to 100%. Shown is the arithmetic mean ± S.E.M. of three independent experiments. * : p < 0.05 (Student’s T-Test). **(C**-**F)** 293T cells were cotransfected with an expression vector for the indicated furin-dependent glycoproteins (1.3 μg) and vector or pcDNA6.90K-myc (1.3 μg). Two days post transfection, cells were lysed and proteins were subjected to Western Blotting using the indicated antibodies. Depicted are the efficiencies of precursor processing, measured by Infrared imaging-based quantification of the respective precursor and the cleavage product.

Defective maturation of *de novo* HIV-1 Env may interfere with its subcellular transport towards the plasma membrane, explaining the paucity of mature HIV-1 Env in released virions. To investigate whether cell surface levels of HIV-1 Env are altered in the presence of 90K, 293T cells were cotransfected with a proviral DNA encoding a GFP reporter gene, and 90K-myc or vector and then subjected to HIV-1 Env surface staining. Surface Env expression was reduced three-fold by 90K (Figure 3A-B)). Importantly, 90K did neither interfere with the subcellular trafficking of another glycoprotein, human CD4, to the cell surface (Additional file 4: Figure S4A-C), nor did it reduce its half-life (Additional file 4: Figure S4D). In contrast, HIV-1 Vpu efficiently prevented accumulation of CD4 at the cell surface and reduced CD4 steady state levels (Additional file 4: Figure S4C-D).

Our results showed that the antiviral activity of 90K involved interference with the proteolytic maturation of HIV-1 Env (Figure 1). During its biosynthesis, the proteolytic cleavage of the HIV-1 Env precursor is mediated by cellular furins and furin-like enzymes residing in the *trans*-Golgi compartment of the infected cell [30]. We thus next analyzed whether 90K may broadly interfere with the proteolytic cleavage of furin-dependent viral and cellular glycoproteins. Similarly to provirally expressed HIV-1 Env, proteolytic processing of CMV promoter driven HIV-1 Env was two-fold less efficient in the presence of 90K (Figure 3C), demonstrating that this effect does not require expression of other HIV-1 proteins. Interestingly, cleavage of the precursor of Ebola GP, preGP, was also impaired (3-fold) by 90K (Figure 3D), whereas proteolytic processing of Influenza virus glycoprotein precursor HA0 and of the precursor of cellular glypican-3, two prototypic furin substrates, was less or not significantly influenced (Figure 3E-F).

Although sensitivities of the furin substrates to physiologic furin concentrations may differ, these results do not provide evidence for a global interference of 90K with furin-mediated cleavage of protein precursors and suggest a specific effect on selected viral glycoproteins. Alternatively, 90K may interfere with HIV-1 Env’s biosynthesis by trapping it in the ER, thus hampering the accessibility of the HIV-1 Env precursor to the furin-positive *cis*-Golgi compartment. We thus probed a direct interaction of HIV-1 Env and 90K. Multiple efforts to immunoprecipitate HIV-1 Env by anti-90K antibodies or *vice versa* did not provide evidence for a direct interaction of both proteins, despite colocolization of HIV-1 Env and 90K-myc (Additional file 5: Figure S6A-B). In contrast, direct interaction of HIV-1 Env and CD4 could be shown (Additional file 6: Figure S5), Deglycosylation tests further failed to disclose any 90K-dependent changes in the subcellular trafficking of HIV-1 Env. Specifically, PNGase treatment efficiently deglycosylated gp160, gp41 and 90K (Additional file 7: Figure S7A). Treatment with endoglycosidase H (EndoH), which cleaves N-linked high-mannose oligosaccharides specifically attached to proteins located in ER/*cis*-Golgi compartment, including HIV-1 gp160 [31,32], deglycosylated gp160 with the same efficiency in the absence and presence of 90K, arguing against 90K retaining a significantly higher percentage of gp160 in the ER (Additional file 7: Figure S7B). In contrast, as expected [32], mature gp41 was resistant to EndoH deglycosylation. These results suggest that 90K-imposed antiviral activity does neither involve interference with furin activity, nor require a direct interaction with HIV-1 Env, nor influence the subcellular transport of the HIV-1 Env precursor from the ER to the *cis*-Golgi.

Conclusively, these results demonstrate that 90K decreases the particle infectivity of newly synthesized HIV-1 particles, probably by interfering with the maturation and incorporation of the viral glycoproteins via a yet to be elucidated mechanism.

### The cell-associated, but not the secreted species of 90K, exerts the antiviral activity

**Figure 4:**
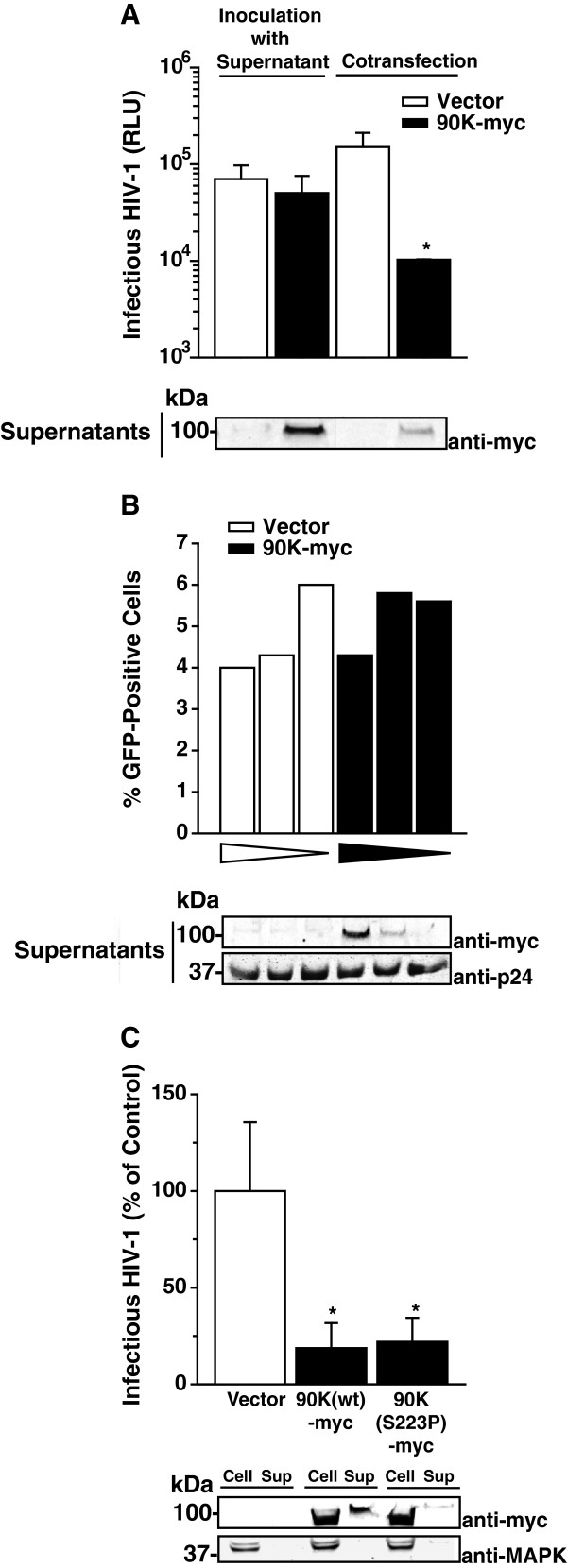
**The cell-associated, but not the secreted species of 90K, exerts the antiviral activity. (A)** 293T cells were either transfected with pBR.HIV-1 NL4.3 IRES GFP and inoculated with culture supernatant originating from vector-transfected or 90K-myc-transfected 293T cells (left half), or cotransfected with HIV-1 wt and pcDNA6.90K-myc or empty vector (right half). Two days post transfection, the amount of infectious HIV-1 in the culture supernatant was determined. Shown are the arithmetic means ± S.D. of triplicates originating from one representative experiment out of two. Supernatants of the cultures from **(A)** were analyzed for 90K-myc content. **(B)** A constant amount of HIV-1 IRES GFP virions was incubated for 15 min at 37°C with decreasing amounts of supernatant derived from 293T cells that had been transfected with empty vector or pcDNA6.90K-myc. TZM-bl were inoculated with the pretreated virus and analyzed for GFP expression three days later. Shown are the values of one representative experiment out of two. An aliquot of the pretreated inoculum that was given to the TZM-bl cells was analyzed by Western Blotting. **(C)** 293T cells were cotransfected with pBR.HIV-1 NL4.3 IRES GFP and indicated expression plasmids. Two days post transfection, the amount of infectious HIV-1 in the culture supernatant was determined. Values are normalized to the empty vector-cotransfected control. Shown are the arithmetic means ± S.E.M. of three independent experiments. Cell lysates and supernatants from one representive experiment were analyzed by Western Blotting. * : p < 0.05 (Student’s T-Test).

By means of its N-terminal signal peptide, the glycoprotein 90K is routed to the extracellular environment via the secretory pathway [26,28]. In order to decipher whether reinternalization of the soluble, secreted species, or the cell-associated form of 90K is responsible for the antiviral effect, we inoculated pHIV-1_NL4.3_-transfected 293T cells with supernatant of 90K-myc-transfected cells or vector-transfected cells (Figure 4A, left half). Regardless of presence or absence of 90K-myc in the supernatant, cells produced comparable levels of infectious HIV-1 (Figure 4A, left half), whereas cotransfection of 90K-myc potently inhibited HIV-1 infectivity. Consistent with this observation, preincubation of HIV-1 IRES GFP virions with 90K-containing supernatants did not affect their infectivity (Figure 4B).

Additionally, we made use of an ER-resident, secretion-impaired 90K-myc mutant, 90K(S223P)-myc [26]. Notably, 90K(S223P)-myc expression inhibited HIV-1 production to a similar extent as wildtype 90K-myc (Figure 4C). We confirmed that this mutant displays a reduced ability to be secreted (Figure 4C). Together, these findings favor a predominant, if not exclusive role of cell-associated 90K in reducing the levels of HIV-1 infectivity, whereas secreted 90K seems to be dispensable for this restricting activity.

### Endogenous 90K reduces the infectivity of HIV-1

**Figure 5:**
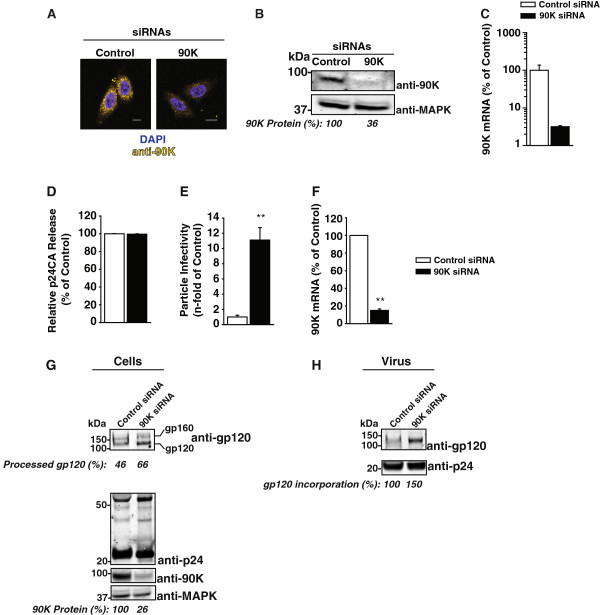
**Endogenous 90K lowers the infectivity of HIV-1. (A)** Confocal microscopy of TZM-bl cells immunostained for endogenous 90K and DAPI. Scale bar: 10 μm. **(B)** Western Blotting analysis of TZM-bl cells using an anti-90K antibody. Values indicate the relative 90K protein expression, as measured by Infrared imaging-based quantification. **(C)** Normalized 90K mRNA levels of the cells shown in **(A)**. **(D)** Following siRNA transfection, TZM-bl cells were infected with VSV-G-pseudotyped HIV-1_NL4.3_ over-night, followed by thorough washing of the cells in order to remove excess virus. Three days post infection, supernatants were analyzed for released p24 capsid antigen and **(E)** particle infectivity, defined as infectivity per ng p24. Bars show the arithmetic means ± S.E.M. of four experiments. **(F)** 90K knockdown efficiencies of the three experiments shown in **(D** and **E)** were validated by quantitative RT-PCR. Bars show the arithmetic means ± S.E.M. **(G)** Lysates of infected cells and **(H)** of sucrose cushion purified viruses from one representative experiment were analyzed by Western Blotting. Percentages indicate the efficiency of gp120 processing, calculated as the signal ratio of gp120 relative to (gp120 + gp160), the relative 90K expression, calculated as the signal ratio of 90K relative to MAPK, and the relative gp120 incorporation, calculated as the amount of gp120 per p24, respectively. **: p < 0.02 (Student’s T-Test).

To examine whether endogenous 90K displays anti-HIV-1 activity, we conducted a siRNA-mediated downmodulation of 90K expression in the HIV-susceptible TZM-bl cell line. This cell line expressed relatively high endogenous levels of 90K, although still a factor of 11.5-fold lower than the level obtained after transfection of 293T cells with the lowest effective 90K-myc plasmid dose (0.15 μg) (Additional file 1: Figure S1B). Transfection of TZM-bl cells with a 90K-specific siRNA potently diminished 90K protein expression as measured by immunofluorescence (Figure 5A), Western Blotting (Figure 5B) as well as 90K mRNA levels (Figure 5C), compared to cells transfected with an irrelevant control siRNA. Following siRNA-mediated downmodulation of 90K expression, TZM-bl cells were infected with VSV-G-pseudotyped HIV-1_NL4.3_. Three days post infection, culture supernatants were analyzed for p24 capsid antigen release and particle infectivity. Importantly, 90K depletion barely affected HIV-1 p24 capsid release (Figure 5D), but boosted the particle infectivity of HIV-1 released into the supernatant by 11-fold compared to control knockdown (Figure 5E), and 90K knockdown was shown to be efficient at the RNA and protein level (Figure 5F-G). Western Blot analysis of producer cell lysates revealed that 90K depletion enhanced the efficiency of gp160 processing, resulting in higher levels of mature gp120 (Figure 5G), whereas HIV-1 p24 capsid expression was unaltered. Sucrose cushion-purified virions displayed increased amounts of incorporated mature gp120 (Figure 5H). Thus, the antiviral potential and mode of action observed for heterologous 90K (Figures 1 and 2) is recapitulated by endogenously expressed 90K, despite low expression levels of endogenous 90K.

### The two central protein-binding domains of 90K are required and sufficient for its anti-HIV-1 activity

**Figure 6:**
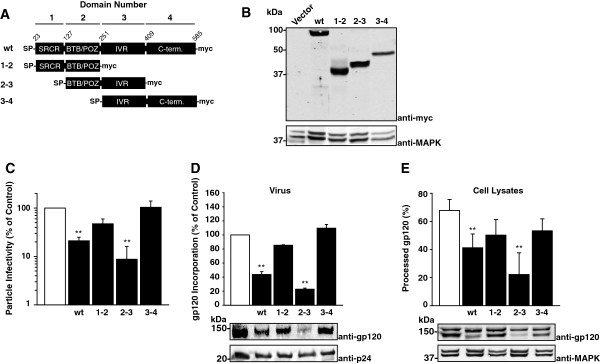
**The two central protein-binding domains of 90K are required and sufficient for the anti-HIV-1 activity of 90K. (A)** Schematic of 90K protein organization and of 90K-myc variants. **(B)** 293T cells were transfected with the indicated constructs, and expression was verified by Western Blotting using an anti-myc tag antibody. **(C)** 293T cells were cotransfected with pBR.HIV-1 NL4.3-IRES GFP and the indicated constructs. Two days post transfection, supernatants were analyzed for particle infectivity, defined as infectivity per ng p24. **(D)** Sucrose cushion-purified virions of three experiments were analyzed by immunoblotting. Shown is the relative gp120 incorporation, as measured by Infrared imaging-based quantification of the amount of gp120 per p24. The signal intensity in absence of 90K expression was set to 100%. **(E)** Cell lysates were analyzed by immunoblotting. Shown is the percentage of processed gp120, as measured by Infrared imaging-based quantification of the amount of gp120 per (gp120 + gp160). The bar diagrams show the arithmetic means ± S.E.M. of six independent experiments. **: p < 0.02 (Student’s T-Test).

90K is composed of a N-terminal scavenger receptor cysteine-rich (SRCR) domain, a BTB/POZ domain, an IVR domain, and a C-terminal part without known homologies to other protein domains [11]. In order to identify domains which are critical for 90K’s antiviral activity, we generated a set of truncated 90K variants which each comprised two domains in their natural order (Figure 6A). All variants were C-terminally myc-tagged, and encoded the authentic signal peptide. Following transient transfection, these variants were expressed at levels similar to those of wildtype 90K-myc, and they displayed the expected respective molecular weight (Figure 6B, [33]). Of note, single domains of 90K were expressed at low to undetectable levels, precluding their use for mutational analysis of 90K’s antiviral activity (data not shown). In order to assess the ability of individual 90K variants to interfere with the infectivity of HIV-1, 293T cells were cotransfected with proviral HIV-1 DNA and the indicated 90K-myc variants or empty vector. Wildtype 90K-myc reduced the particle infectivity of HIV-1 virions released in the culture supernatant, whereas the particle infectivity of HIV-1 remained largely unchanged in the presence of 90K(1,2)-myc and 90K(3,4)-myc (Figure 6C). However, 90K(2,3)-myc was at least as potent in diminishing HIV-1 infectivity levels as wildtype 90K-myc, suggesting that the presence of the BTB/POZ and IVR domains of 90K is required and sufficient for 90K’s antiviral activity. Importantly, 90K(2,3)-myc reduced the incorporation of HIV-1 gp120 even more potently than 90K-myc wildtype, whereas viruses produced in 90K(1,2)-myc and 90K(3,4)-myc expressing cells displayed normal levels of incorporated HIV-1 gp120 (Figure 6D). Furthermore, the proteolytic cleavage of gp160 in cells transfected with 90K(2,3)-myc was inefficient, like in 90K-myc wildtype cells, whereas 90K(1,2)-myc and 90K(3,4)-myc induced only minor changes in the efficiency of Env processing (Figure 6E).

Conclusively, a region within the two intermediate protein-binding domains of 90K, the BTB/POZ and the IVR domain, is required and sufficient for its ability to interfere with HIV-1 Env maturation and incorporation, whereas the scavenger receptor cysteine-rich domain and the C-terminal domain of 90K are dispensable.

### Species specificity of 90K-imposed antiviral activity

Mice encode a 90K homolog, Cyclophilin C-associated protein (CypCAP), which displays 69% homology on the amino acid level [34]. Human 90K and murine CypCAP share the protein organization and the ability to get secreted [35]. Of note, rodent cells display severe limitations in supporting the late phase of HIV-1 replication, including virion assembly and infectivity [36], hampering the development of an immunocompetent small animal model of HIV infection.

We thus tested the ability of mouse CypCAP to reduce the particle infectivity of progeny HIV-1. Remarkably, the mouse homolog of 90K failed to act antivirally, whereas human 90K reduced the particle infectivity of HIV-1 (Additional file 8: Figure S8A). The lack of functional inhibition by mouse CypCAP was accompanied by normal Env glycoprotein incorporation (Additional file 8: Figure S8B). Although we observed a trend towards slightly decreased processing of the gp160 precursor, the difference was not statistically significant (Additional file 8: Figure S8C). Thus, these data suggest that the negative modulations of human 90K on Env maturation and virion incorporation are required for its ability to reduce HIV-1 particle infectivity. Furthermore, CypCAP does not seem to contribute to the inefficiency of late steps of the HIV-1 replication cycle in murine cells.

### Expression and susceptibility of *90K* to IFN stimulation in primary HIV-1 target cells

**Figure 7:**
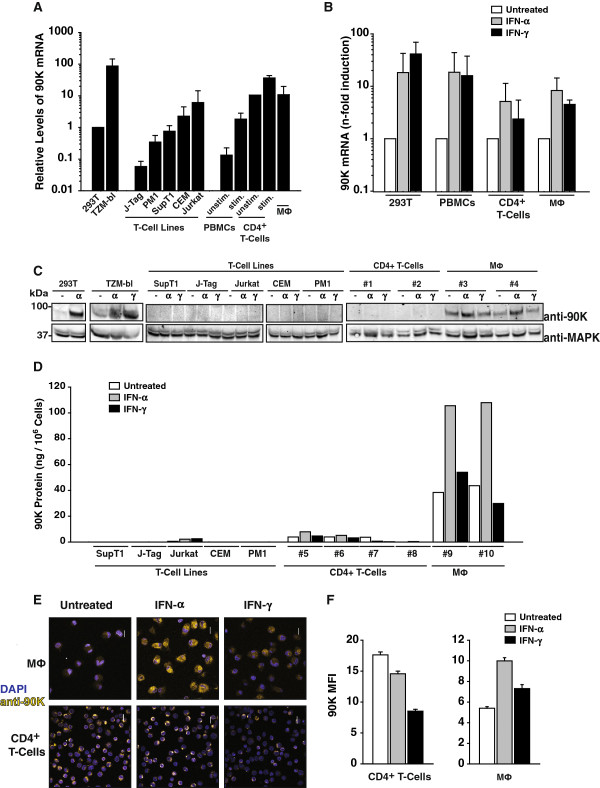
**Expression and susceptibility to IFN stimulation of *90K *in primary HIV-1 target cells. (A)** cDNA was prepared from total RNA of the indicated cell lines and primary cells, and analyzed for relative *90K* mRNA expression by TaqMan-based quantitative PCR with *RNaseP* expression as a reference. Depicted is the relative level of *90K* expression, and values obtained for 293T cells were arbitrarily set to 1. Histograms represent arithmetic means ± S.E.M. of three independent experiments, except for the data for CD4^+^ T-cells, which shows arithmetic means of two experiments. **(B)** Indicated primary cell cultures were treated with IFN-α (100 U/ml) or IFN-γ (100 U/ml) for 24-48 hours, or left untreated, prior to RNA isolation and cDNA synthesis. Shown is the fold increase of 90K mRNA level from 2-3 experiments, each performed in duplicates. **(C)** Western Blot analysis of indicated cells treated with IFN-α (100 U/ml) or IFN-γ (100 U/ml) for 48 hours, or left untreated. **(D)** Anti-90K ELISA of cell lysates, following treatment with IFN-α (100 U/ml) or IFN-γ (100 U/ml) for 48 hours where indicated. **(E)** Immunofluorescence stain of indicated primary cell cultures. Cells treated with IFN-α (100 U/ml) or IFN-γ (100 U/ml) for 24-48 hours where indicated or left untreated. Scale bar: 10 μm. **(F)** Quantification of 90K immunofluorescence by KNIME image processing plug-in and the mean fluorescence intensity (MFI) signal per cell was determined. The data represent the arithmetic mean ± S.E.M. of 154 untreated, 259 IFN-α-treated and 147 IFN-γ-treated T-cells and the mean of 85 untreated, 121 IFN-α-treated and 64 IFN-γ-treated macrophages.

90K protein has been reported to be expressed in many tissues and tumor cell lines [11], but whether it is expressed in CD4^+^ T-cells and macrophages that represent the primary HIV-1 target cells *in vivo* is poorly investigated. Thus, we examined endogenous 90K mRNA and protein expression in these cell types. 90K mRNA was expressed in primary HIV-1 target cells, including activated PBMCs, CD4^+^ T-cells and monocyte-derived macrophages (Figure 7A). Notably, IL-2/mitogen stimulation increased 90K messenger levels in PBMCs and CD4^+^ T-cells. In line with the documented IFN-responsiveness of 90K mRNA expression [14,15], treatment of primary PBMCs, stimulated CD4^+^ T-cells, and monocyte-derived macrophages with IFNs resulted in a marked enrichment of 90K transcripts (Figure 7B).

We next assessed the relative levels of 90K protein expression in HIV-1 target cells. Western Blot analysis failed to reproducibly reveal basal expression levels of 90K in multiple T-cell lines, activated PBMCs (data not shown) and CD4^+^ T-cells (Figure 7C), despite detection of 90K mRNA (Figure 7A), whereas all tested macrophages scored positive (Figure 7C). IFN-α, and, to a lesser extent, IFN-γ treatment enhanced 90K protein levels in macrophages (Figure 7C). Both IFN-α and IFN-γ treatment failed to induce 90K protein expression in CD4^+^ T-cells to levels detectable by Western Blotting.

In order to verify the findings on 90K expression obtained by Western Blotting, we made use of a sensitive commercial anti-90K antigen ELISA for analysis of cell-associated 90K using lysates of T-cell lines and primary HIV-1 target cells. This assay revealed that 90K levels were close to the detection limit in Jurkat T-cells and CD4^+^ T-cells, whereas the other T-cell lines scored negative. In macrophages, screening of macrophages from multiple donors by anti-90K ELISA confirmed that this cell type expresses higher 90K protein levels than T-cells.

Immunofluorescence analysis revealed, intuitively intriguingly, higher 90K staining intensities in CD4^+^ T-cells than macrophages (Figure 7E, middle and bottom panels, and Figure 7F). However, a direct comparison of the 90K signals obtained in CD4^+^ T-cells and macrophages by immunofluorescence seems inadequate, since macrophages and T-cells may greatly differ in their sensitivity to Triton permeabilization and thus to effective intracellular antibody concentrations. The intra-cell type comparison of IFN-mediated upregulation, however, confirmed that IFN-α increased 90K expression in macrophages, but not in CD4^+^ T-cells. Together, 90K is expressed in macrophages and, at low levels, in CD4^+^ T-cells.

### Reduction of endogenous 90K expression enhances the particle infectivity of HIV-1, increases HIV-1 Env content of progeny virions and accelerates viral spread in primary HIV-1 target cells

**Figure 8:**
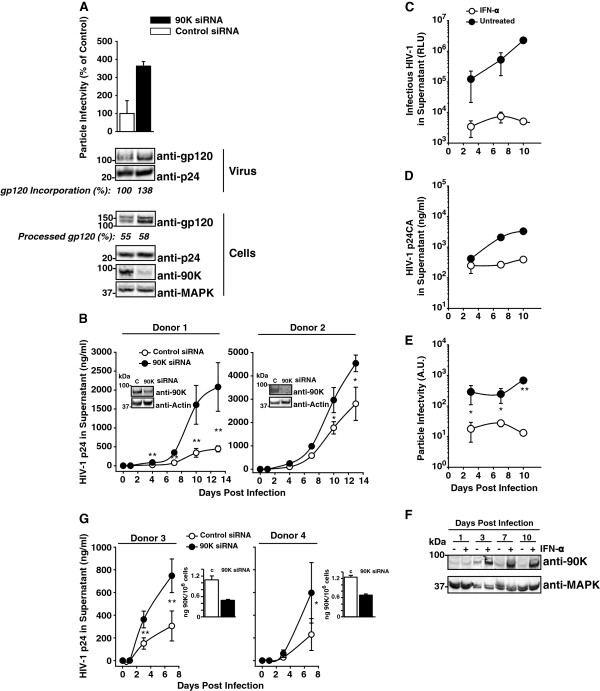
**Depletion of 90K in primary HIV-1 target cells enhances particle infectivity, enhances Env content of HIV-1 particles, and accelerates HIV-1 spread. (A)** VSV-G/HIV-1 _NL4.3_-infected macrophages were transfected one day post-infection with a 90K-specific or an irrelevant siRNA. At day 7 post infection, particle infectivity of released HIV-1 was calculated as the ratio of infectivity to p24 capsid antigen. Cell lysates and sucrose cushion-purified virus were analyzed by Western Blotting. Percentages indicate the relative gp120 incorporation and the efficiency of gp120 processing, calculated as in Figure 5**(G**-**H) (B)** Macrophages obtained from two donors were siRNA-transfected and subsequently infected with HIV-1_Ba-L_. One day post infection, excess virus was removed by thorough washing. Supernatants were subjected to anti-p24 capsid antigen ELISA at indicated time points. Aliquots of cells were taken for 90K knockdown validation over time by Western Blotting. Shown is 90K expression at day 13 post infection. **(C)** Macrophages were infected with HIV-1_Ba-L_ and simultaneously treated with IFN-α (100 U/ml). Supernatants were harvested at the indicated time points, frozen, and analyzed for released p24 capsid and **(D)** infectious HIV-1. Shown are arithmetic means of triplicates ± S.D. **(E)** Using the data from **(C)** and **(D)**, the particle infectivity was calculated. Data points indicate arithmetic means of triplicates ± S.D. **(F)** Cell lysates were subjected to Western Blotting using indicated antibodies. **(G)** IL-2/PHA-stimulated PBMCs from two donors were nucleofected with a 90K-specific or an irrelevant siRNA followed by HIV-1_NL4.3_ infection over night. Excess virus was removed by thorough washing at indicated time points supernatants were subjected to p24 capsid antigen ELISA. 90K knockdown at the time point of infection was validated by 90K ELISA. * : p < 0.05; **: p < 0.02 (Student’s T-Test).

Basal 90K levels in primary macrophages were up to 9-fold lower than levels obtained by transfection of 293T cells with the lowest effective 90K-myc plasmid dose (0.15 μg) (Additional file 1: Figure S1C). In order to assess the antiviral potential of these low endogenous 90K expression levels in this primary HIV-1 target cell type, we infected primary macrophages with VSV-G/HIV-1_NL4.3_ and subsequently reduced 90K expression by RNAi. Virions purified from culture supernatants of infected macrophages were examined for their particle infectivity and for their protein composition. Particle infectivity was enhanced by 90K depletion (Figure 8A), and levels of HIV-1 Env were increased in virions produced by 90K-depleted macrophages compared to virus which originated from control siRNA-transfected cultures, corroborating 90K’s ability to induce a paucity of mature Env in virions (Figure 8A). Analysis of corresponding cell lysates showed that the efficiency of HIV-1 Env processing was only mildly increased, suggesting that 90K’s primary mode of action in macrophages is the inhibition of virion incorporation of HIV-1 Env, rather than inhibition of Env biosynthesis. We next tested whether 90K depletion influences HIV-1 spread in primary macrophages. For this purpose, macrophages from two donors were transfected with a 90K-specific or a control siRNA and subsequently infected with macrophage-tropic HIV-1_Ba-L_. Supernatants were analyzed for p24 capsid amounts released in the culture supernatant as a result of HIV-1 spread. Notably, silencing of 90K expression was stable and resulted in an enhanced spread of HIV-1_Ba-L_ (Figure 8B), showing that low endogenous levels of 90K protein can impose a hurdle to HIV-1 replication.

Given the *90K* upregulation following IFN-α stimulation in primary macrophages (Figure 7B-F), we next tested whether IFN-α treatment translates into a lower particle infectivity of HIV-1 produced in this primary cell type. For this purpose, we infected macrophages with HIV-1_Ba-L_ in the presence or absence of a continuous and moderate dose of IFN-α (100 U/ml). IFN-α treatment potently reduced the levels of released HIV-1 infectivity (Figure 8C) and, to a lower extent, of released p24 antigen (Figure 8D), probably involving tetherin-mediated inhibition of virus release [2,3]. At days 3, 7 and 10 post initiation of IFN-α treatment and HIV-1 infection, the particle infectivity of HIV-1 was severely decreased by IFN-α treatment compared to no treatment (Figure 8E). Western Blotting of cell lysates demonstrated potent induction of 90K expression at these time points, but not at day 1 post initiation of IFN-α treatment and HIV-1 infection, probably due to too short duration of IFN stimulation (Figure 8F). Due to IFN-α-mediated upregulation of multiple genes, these results do not specifically address 90K’s contribution to the IFN-α-mounted antiviral response. However, they indicate the presence of IFN-α-induced blocks to particle infectivity and suggest that 90K may constitute a part of this antiviral defense response.

Finally, we tested whether low levels of 90K expression in primary PBMCs are sufficient for influencing HIV-1 replication. A two-fold reduction of 90K expression resulted in a modest, but clearly detectable acceleration of HIV-1 spread in PBMC cultures infected with T-cell tropic HIV-1_NL4.3_.

Together, these findings establish that, although low, endogenous levels of 90K in primary HIV-1 target cells induce a paucity of mature Env molecules in newly synthesized HIV-1 particles, reduce the infectivity of HIV-1 and impair HIV-1 spread. These findings highlight the relevance of this antiviral factor in physiologic target cell types of HIV-1 infection.

## Discussion

Although the transcription of many IFN-stimulated genes is induced upon contact of pathogens with the host cell, the direct contribution of most of their respective gene products to the mounting of an antiviral state is poorly investigated. Here, we propose that 90K is a player of the intrinsic immune response that may contribute to HIV-1 restriction.

Two hallmarks of antiviral restriction factors are their ability to directly and dominantly inhibit specific steps of the virus replication cycle, and their IFN-induced or IFN-stimulated expression. These criteria are fulfilled by 90K. First, heterologous expression and siRNA-mediated depletion studies demonstrated that 90K reduces the particle infectivity of HIV-1, without interfering with particle release, expression of other viral proteins, or inducing detectable toxicity. Both heterologous 90K as well as endogenous 90K, expressed in the HIV-1-susceptible TZM-bl cell line and, most importantly, in primary macrophages and primary PBMCs, were antiviral. Reduction of particle infectivity by 90K coincided with inefficient proteolytic cleavage of the gp160Env precursor, reduced cell surface expression of HIV-1 Env, and limited incorporation of gp120 and gp41 into progeny virions.

Second, we and others [14-16] clearly demonstrate that 90K expression can be induced or stimulated by IFN-α. Interestingly, 90K protein, but not mRNA, induction seems to be governed by cell type specific regulation. 293T cells and macrophages, but not T-cells, were susceptible to IFN-α-mediated induction and stimulation of 90K protein expression, respectively. In contrast, T-cells, in addition to displaying low to undetectable levels of 90K expression, were refractory to IFN-mediated upregulation of 90K protein expression. A recent study suggested that T-cells and macrophages, although equally responsive to IFN-α in terms of ISG mRNA upregulation, differ in their ability to mount an antiviral state upon IFN-α-treatment, suggesting that ISG mRNA induction may not reflect the degree of protein upregulation in T-cells [37]. Future studies are required to study in depth the regulation of 90K protein expression in T-cells.

Physiological levels of 90K in HIV-1-susceptible TZM-bl cells, primary macrophages and PBMCs were clearly lower than ectopic antiviral 90K expression levels. Despite this fact, the limited level of endogenous 90K expression clearly was able to diminish the particle infectivity of HIV-1, since siRNA-mediated depletion of endogenous 90K in TZM-bl cells and primary macrophages resulted in an ameliorated particle infectivity of released virions and enhanced Env incorporation. In contrast, ectopic 90K protein seems to display, in relative terms, weak antiviral activity. Importantly, 90K knockdown resulted in accelerated spread of HIV-1 in primary macrophages and PBMCs. Interestingly, an IFN-induced loss of particle infectivity of newly synthesized HIV-1 particles has been described in PHA/IL-2-stimulated PBMCs [38] and myeloid U937 cells [39]. In these studies, the reduction of infectivity was attributed to an aberrant distribution of Env in intracellular compartments and a marked depletion of envelope glycoproteins in released virions. It is plausible that these effects are, at least partially, caused by 90K.

Since HIV-1 Env and 90K share, by means of their N-terminal signal peptide, subcellular trafficking through the secretory pathway, it was tempting to speculate that a direct or indirect association of these two proteins is required for 90K’s restricting activity. HIV-1 gp160Env molecules require proteolytic cleavage by cellular furin and furin-like enzymes located in the *trans*-Golgi network in order to reach fusogenicity. It is unlikely that ectopic 90K expression globally interferes with the furin-mediated cleavage of glycoprotein precursors or with the subcellular trafficking of glycoproteins to the cell surface, since neither the processing efficiency of two other furin substrates, the precursors of FPV HA0 and of cellular glypican-3, nor the subcellular transport of cellular glycoprotein CD4 to the plasma membrane were grossly influenced by 90K. Of note, the HA0 precursor protein studied here is derived from the H7 HA of Influenza virus and displays a polybasic cleavage site (KKRKKR) which is highly efficiently cleaved by furin and may be less sensitive to modest changes of furin efficacy. HIV-1 Env, in contrast, has been reported to be a notoriously poor furin substrate [40] and might require much higher furin levels than the prototypic furin substrates HA and Glypican-3. Future goals will consist to probe direct or indirect interaction of furin and 90K. Notably, however, the proteolytic processing of the precursor of Ebola GP was severely impaired in the presence of 90K, suggesting that 90K may reduce the particle infectivity of Ebola virus.

We next hypothesized that 90K may prevent access of the unprocessed gp160 precursor to the furin-positive *trans*-Golgi compartment by a direct interaction, resulting in an abolished proteolytic cleavage and, as a consequence, in a reduced incorporation of mature gp120 into budding particles. This scenario, however, could not be substantiated by deglycosylation experiments and immunoprecipitation assays. These data, however, do not exclude an indirect association of 90K and HIV-1 Env within a multi-protein complex. Mutational analysis revealed that the two central protein-binding domains, BTB/POZ and IVR, are crucial for 90K’s antiviral activity, whereas the SRCR domain and the carboxyterminal domain of 90K were dispensable. Future work will consist to determine the exact mechanism of 90K-imposed inhibition.

Interestingly, the mouse homolog of 90K, CypCAP, failed to act antivirally. Cyclophilins catalyze petidyl-prolyl *cis-trans* isomerase reactions and assist protein folding [41]. Whether antiviral human 90K likewise modulates chaperone activity and whether this is required for exerting the antiviral effect is unknown. Importantly, the inactivity of the mouse protein was accompanied by absent alterations of gp120 incorporation and processing, suggesting that these two effects are crucial for human 90K’s ability to reduce the particle infectivity of HIV-1. However, 90K’s effect on gp120 processing was at best mild in primary macrophages, whether virion incorporation of Env was clearly increased, suggesting that inhibition of Env processing and virion incorporation are not necessarily coupled.

In healthy individuals, 90K is present in multiple body compartments, including blood, semen, urine, tears, saliva, and breast milk [18,27,28]. Despite of its designated location in the extracellular milieu, we found that not the secreted species, but rather the cell-associated 90K exerted the reduction of particle infectivity. However, that does not exclude that secreted 90K displays antiviral functions *in vivo* that may depend on its reported immunostimulatory functions [11]. In line with this possibility, lack of mother-to-child transmission of HIV-1 correlated with elevated serum levels of 90K in HIV-1 infected mothers [42]. Also, elevated levels of 90K in breast milk were reported to induce protective effects against acute respiratory infections in breast milk fed children [27,43]. Furthermore, 90K was recently described as a ligand of DC-SIGN and may interfere with the capturing of virions that may be required for the *trans*-infection of T-cells [44] at sites of primary infection.

A third criterion of many restriction factors is the existance of virally encoded antagonists against them, expressed either by HIV-1 or HIV-1 precursors. Work of our laboratory has provided preliminary evidence for the presence of an HIV-1 encoded antagonist of human 90K (unpublished observation), yet the specificity of the observed antagonism is not established and requires substantial experimental proof. Of note, low density of glycoprotein molecules on the virus surface is a specific property of HIV-1, which has been suggested to provide a selective advantage. Specifically, it has been hypothesized that this feature may enable efficient escape from antibody-mediated immune response [45]. It is unclear whether the reduction of infectivity, induced by 90K-mediated paucity of Env in virions, is outweighed by the advantage of better immune system escape. Future studies are required to determine the net antiviral effect of 90K *in vivo*, as well as possible viral evasion strategies that may circumvent 90K-imposed antiviral activity.

## Conclusions

This study establishes 90K as an antiviral factor which impairs the infectivity of HIV-1 progeny, involving defective maturation and incorporation of Env glycoproteins. Further investigation and exploitation of its mode of action may provide a platform for the design of novel therapeutic antiviral approaches and widen our understanding of cell-mediated antiviral defense mechanisms.

## Methods

### Cells

293T cells, TZM-bl cells and the T-cell lines SupT1, JTag, PM1, CEM, Jurkat were cultured as recommended by ATCC. Cultures of primary PBMCs, from random human donors were generated as previously reported [46]. Taking of blood samples from humans and cell isolation were conducted with approval of the local ethics committee (Ethik-Komission der Universität Ulm, approval No. 298/12). Human blood samples were taken from healthy blood donors, who provided written informed consent for the collection of samples and subsequent cell isolation and analysis. Macrophages were differentiated for 10-15 days in the presence of 10% human AB serum (Sigma). CD4^+^ T-cells were isolated from Buffy Coat by negative selection with the RosetteSep Human CD4^+^ T Cells Enrichment Cocktail (StemCell Technologies). For the 90K expression studies, CD4^+^ T-cells were stimulated with IL-2/PHA for 2-3 days prior to IFN-α (Roche) or IFN-γ (Sigma-Aldrich) treatment. 293T cells were transfected by calcium phosphate DNA precipitation, SupT1 cells were transfected by electroporation.

### Plasmids

pcDNA6 and pcDNA6.90K-myc plasmids were provided by Ji Hee Lee, South Korea. pIRES2 90K-myc IRES GFP was generated by PCR amplification of 90K-myc and insertion into pIRES2-GFP (Clontech) using *Nhe*I and *Bam*HI. The truncated variants of 90K-myc all contained the authentic signal peptide and were generated by amplification of 90K with the following primers using *Hind*III and *Xba*I and insertion into pcDNA6: fwd GCAAGCTTGGATGACCCCTCCG and rev CGTCTAGAGGGGAGGAGGATGGCAAAGAGGCTTGC (90K(1,2)-myc); fwd CGAAGCTTATGACCCCTCCGAGGCTCTTCTGGGTGTGGCTGCTGGTTGCAGGAACCCAAGGCGTGAACGATGGTGACACCAGGAGCACCCACACCCTGGACCTC and rev CGTCTAGAAATCCGGGGCTTGTAGGTATCCT (90K(2,3)-myc); fwd CGAAGCTTATGACCCCTCCGAGGCTCTTCTGGGTGTGGCTGCTGGTTGCAGGAACCCAAGGCGTGAACGATGGTCTCCTCCCCCAGGACCCCTCGTTCCAGATG and rev CCTCTAGAGTCCACACCTGAGGAGTTGGTCAGG (90K(3,4)-myc). pcDNA6-mouse CypCAP-myc was generated by amplification of the mouse cDNA (Sino Biological Incorportion) using primers carrying *Hind*III and *Xba*I restriction sites (fwd GCAAGCTTATGGCTCTCCTG and rev GCTCTAGACACCATGTCAGTG) and ligation of the amplicon into pcDNA6/myc. Expression constructs for Glypican-3-HA and H7 Influenza virus HA (derived from A/chicken/Rostock/8/1934(H7N1)) were provided by Guido David and Wolfgang Garten, respectively. The expression plasmid pEbola GP was from Mark Goldsmith. The CD4 and the HIV-1_NL4.3_ Env expression plasmid was from Oliver Keppler. The Vpu-IRES GFP expression construct and the respective control vector have been described elsewhere [47].

### Viruses

Infectious HIV-1 was generated by calcium phosphate DNA precipitation of 293T cells with plasmids encoding HIV-1 and harvest of cell-free virion-containing supernatant. Plasmids encoding NL4.3, LAI, YU-2 were obtained from Oliver Keppler, plasmids encoding transmitter founder viruses (CHO58, THRO, CHO77) were obtained from Frank Kirchhoff. The construct pBR.HIV-1 NL4.3-IRES GFP carries an IRES-EGFP cassette downstream of the *nef* ORF [48]. The expression plasmid for VSV-G was provided by Oliver Keppler. HIV-1 Ba-L was passaged on PM-1 cells.

### HIV-1 infectivity assay

The infectivity of HIV-1 virions was assessed by applying a standardized TZM-bl based firefly luciferase assay or beta-galactosidase assay. Readouts were obtained luminometrically (RLU, relative light units). For calculation of the particle infectivity, luminometric counts were divided by the content of p24.

### Anti-p24 antigen ELISA

Supernatants were analyzed for p24 content (ng/ml) by applying a home-made anti-p24 antigen ELISA [49].

### Cell viability assays

Cells were stained with 7-AAD for detection of dead or unhealthy cells (BD PharMingen). For measuring metabolic activity, cell lysates were analyzed using the Cell Titer Glow assay (Promega). As a positive control for apoptosis induction or cell death, cells were treated UV-irradiated.

### SiRNAs and siRNA-mediated depletion of 90K expression

The following siRNAs were used: control siRNA (Dharmacon), 90K-specific siRNA (GAAGCUCUGCCUACAGUUC, from MWG). TZM-bl cells and primary macrophages were transfected 2-3 times with siRNAs at the final concentration of 5-15 nM, using RNAiMax (Invitrogen). PBMCs were transfected twice by Amaxa nucleofection (Lonza) with final siRNA concentration of 2.5 μM. Knockdown efficiency was determined by quantitative RT-PCR of 90K mRNA and RNaseP mRNA and/or by anti-90K Western Blotting or anti-90K ELISA (eBioscience).

### 90K mRNA analysis

Total RNA extraction from untreated or IFN-treated cells was performed with the Qiagen RNeasy kit (Qiagen), followed by DNase treatment (Ambion) and cDNA synthesis (NEB, Invitrogen). Quantification of relative 90K mRNA levels was performed with the Step One Plus Real Time PCR System (Applied Biosystems) using TaqMan PCR technology with the following oligonucleotide primers: 5′-GCTTCCTTCCTCTCTGCAATGA-3′ (forward primer), 5′-TCAGGTGAGTAGGGCGACATC-3′ (reverse primer), 5′-FAM-CTTCAACAACCGGCCAC-TAMRA-3′ (fluorescent probe). Relative 90K mRNA levels were determined using the ∆∆Ct method with human *RNaseP* mRNA (Applied Biosystems) as internal reference. Each sample was analyzed in duplicates. Data analysis was performed using Applied Biosystems Step One Software v2.1.

### Confocal immunofluorescence microscopy

Cells grown on coverslips were transfected with Lipofectamin2000 (Invitrogen) and fixed with 4% paraformaldehyde. They were permeabilized with 0.2% Saponin or 0.1% TritonX-100, blocked and stained with goat polyclonal anti-90K (R&D Systems), followed by anti-goat Alexa647, goat polyclonal anti-myc (novus), followed by anti-goat Alexa488, or Chessie8 Hybridoma supernatant, followed by anti-mouse Alexa647. Coverslips were mounted with Mounting medium containing DAPI (Vectashield) and analysed with a Zeiss 710 LSM confocal microscope. Images were recorded and processed with the ZEN (2010) software. Quantification of 90K expression was performed by using the open-source KNIME (Kontanz Information Miner) platform (see http://tech.knime.org/faq#q1_1) in combination with the KNIME Image Processing 1.0.3 extension. For segmentation, DAPI stained nuclei were used. The images were smoothed using a Gaussian Filter followed by a simple Otsu thresholding. Consecutively, cells were splitted using a standard cell clump splitter and additionally filtered by size. After identification of the cells, the intensity of each pixel was measured, the mean of intensity for each cell was calculated.

### Antibodies

The following antibodies/antisera were used: goat polyclonal anti-90K (R&D Systems), mouse anti-myc (provided by Jens von Einem), goat polycolonal anti-myc (Novus Biologicals), rabbit anti-MAPK (Santa Cruz), mouse anti-HIV-1 p24 (Exbio), rabbit anti-HIV-1 gp120 and mouse anti-HIV-1 gp41 (provided by Valerie Bosch), human 2G12 (NIH), mouse anti-HA (abcam), anti-Ebola GP (provided by Stephan Becker), anti H7-serum (provided by Wolfgang Garten), rabbit anti HIV-1 Vpu (provided by Ulrich Schubert), mouse anti-CD4 (abcam), anti-CD4-APC (BD).

### Immunoblotting

Cells were lysed with M-PER Mammalian Protein Extraction Reagent (Pierce), and virions were prepared from supernatants and ultracentrifugation through a 20% (w/v) sucrose cushion. Proteins were run on a 12.5% or 7.5% SDS-PAGE and transferred onto nitrocellulose. Blocked membranes were incubated with primary antibodies. Secondary antibodies conjugated to Alexa680/800 fluorescent dyes were used for detection by Odyssey Infrared Imaging System (LI-COR Biosciences) and quantification by Odyssey software.

### Coimmunoprecipitation

Cells were lysed with 0.5% NP40 lysis buffer and a small aliquot was used for control of protein expression (Input). Residual lysate was filled up to final volume of 500 μl with 0.5% NP40 and incubated with antibody for 1 hour at 4°C. 30 μl G-Sepharose Beads (GE-Healthcare) were added and precipitated 90 minutes at 4°C. Beads were washed with 0.1% NP40, treated with Western Blot Sample Buffer and boiled for 5 minutes. Supernatants contained the precipitated proteins.

### Digestion with PNGase or EndoH

Cell lysates were treated with PNGase (NEB BioLabs) and EndoH (NEB BioLabs) according to instructor’s protocol. Digestion was performed with 625 Units PNGase for 20 minutes or with 100 Units EndoH for 5 minutes at 37°C.

## Abbreviations

A.U: Arbitrary units; HIV: Human immunodeficiency virus; IFN: Interferon; LGALS3BP: Lectin galactoside-binding soluble 3 binding protein; MFI: Mean fluorescence intensity; RLU: Relative light units.

## Competing interests

The authors declare that they have no competing interests.

## Authors’ contributions

VL and CG conceptualized and designed the study; VL, KS, NS, CK, CMS, TW, DK, CG performed the study; TW and CD performed the immunofluorescence-based quantification of 90K expression; VL, KS, CK, CMS, CG analyzed data; VL and CG wrote the paper. JM and FK provided reagents. All the authors reviewed the manuscript and approved the final version.

## Supplementary Material

Additional file 1: Figure S1 Heterologous 90K levels compared to endogenous 90K levels. (A) 293T cells were cotransfected with various amounts of pcDNA6.90K-myc (1.3, 0.4, 0.15, 0.05, 0.02 μg), stimulated with 100 Units IFN-α, IFN-γ for 48 h, or left untreated. (B) Western Blot of cell lysates of 293T cells overexpressing various amounts of 90K-myc (A) and untreated TZM-bl cells. (C) Western Blot of cell lysates of 293T cells overexpressing various amounts of 90K-myc (A) and primary macrophages stimulated with 100 IFN-α, IFN-γ for 48 h, or left untreated. The numbers depict the relative 90K expression, measured by normalization of the 90K signal to the MAPK signal obtained by Infrared-imaging based quantification. The ratio obtained for the lowest antivirally active 90K plasmid dose (0.15 μg, A), the ratio obtained for TZM-bl cell lysates (B) or the ratio obtained for untreated macrophages (C) was set to 1, respectively. Click here for file

Additional file 2: Figure S2 90K reduces the particle infectivity of multiple HIV-1 strains. (A) 293T cells were cotransfected with the indicated proviral plasmids and highest amount of pcDNA6.90K-myc or empty vector. Supernatants were analyzed for infectious HIV-1 using a luminometric TZM-based luciferase assay. Shown are the results of one representative experiment out of three-six. (B) Relative levels of particle infectivity, defined as HIV-1 infectivity per ng p24 capsid are depicted. (C) Sucrose cushion-purified virions were analyzed by immunoblotting. Percentages indicate the relative gp120 incorporation, as measured by Infrared imaging-based quantification of the amount of gp120 per p24. The signal intensity in absence of 90K expression was set to 100%. (D) Cell lysates were analyzed by immunoblotting using the indicated antibodies. Numbers indicate the efficiency of gp160 processing. * : p < 0.05; **: p < 0.02 (Student’s T-Test). Click here for file

Additional file 3: Figure S3 90K-myc expression is not associated with toxicity or reduced metabolic activity. (A) 293T cells were transfected with pcDNA6.90K-myc (1.3 μg), empty vector, or UV-irradiated and stained two days post transfection and one day post UV-irradiation with 7-AAD. Shown are representative dot plots of one experiment out of two. Numbers indicate percentage of 7-AAD-positive cells. (B) Quantification of 7-AAD FACS analysis. (C) Cells were lysed and analysed for metabolic activity by Cell Titer Glow assay. Shown are the RLU of triplicates obtained from one representative experiment out of two. Click here for file

Additional file 4: Figure S4 90K does not reduce the cell surface levels of CD4. (A) 293T cells were cotransfected with pcDNA.CD4 and pIRES2EGFP.90K-myc or empty vector, Cells were stained with APC-conjugated anti-CD4 and analyzed by flow cytometry. Shown are representative dot plots of one experiment out of three. (B) 293T cells were cotransfected with pcDNA.CD4 and pVpu-IRES GFP or empty vector and processed like in (A). (C) CD4 cell surface levels were calculated by comparing, within the same sample, CD4 levels on non-GFP-expressing cells (gate P2) with CD4 levels on cells with medium-high GFP expression levels (gate P3). CD4 levels on vector transfected cells were set to 100%. (D) An aliquot of the cells shown in (A) and (B) were lyzed and analyzed by Western Blotting using the indicated antibodies. Click here for file

Additional file 5: Figure S5 90K and Env colocalize to a high extent. (A) 293T cells were cotransfected with pcDNA6.90K-myc and an HIV-1 Env expression plasmid, and stained for 90K-myc (green) and Env (red). Scale bar: 10 μm. (B) The classic colocalization coefficient was calculated for the colocalization of 90K protein with Env protein or *vice versa* using ZEN2010 software. The data represent the arithmetic mean ± S.D. of 105 analyzed cells. Click here for file

Additional file 6: Figure S6 No evidence for a direct interaction of 90K and HIV-1 Env. (A-C) 293T cells were cotransfected with pcDNA6, pcDNA6.90K-myc, an HIV-1 Env expression plasmid, pcDNA.CD4 or a combination out of these. (A) 90K, CD4 and bound proteins were precipitated from cell lysates by an anti-90K or anti-CD4 antibody, respectively. (B) 90K, CD4 and bound proteins were precipitated from cell lysates by an anti-myc or anti-CD4 antibody, respectively. (C) Env and bound proteins were precipitated from cell lysates by an anti-gp120 antibody. For each experimental set up, an aliquot of whole cell lysate for expression control (Input) and the precipitated proteins were analyzed by Immunoblot with indicated antibodies. Click here for file

Additional file 7: Figure S7 90K does not retain Env in the ER. (A-B) 293T cells were cotransfected with pBR.HIV-1 IRES GFP and vector or pcDNA6.90K-myc. (A) Cell lysates were treated with PNGase. (B) Cell lysates were treated with EndoH. Deglycosylated and control proteins were analyzed by Western Blot. Numbers indicate the efficiency of gp41 processing, calculated as the signal ratio of gp41 relative to (gp41 + gp160), or the percentage of deglycosylated gp160 to the total gp160 signal. Click here for file

Additional file 8: Figure S8 Species specificity of 90K-imposed antiviral activity. (A) 293T cells were cotransfected with pBR.HIV-1 NL4.3-IRES GFP and 1.3 μg of the indicated expression constructs or empty vector. Two days post transfection, supernatants were analyzed for particle infectivity, defined as infectivity per ng p24. Cell lysates were analyzed by immunoblotting. (B) Sucrose cushion-purified virions were analyzed by immunoblotting. Shown is the relative gp120 incorporation, as measured by Infrared imaging-based quantification of the amount of gp120 per p24. The signal intensity in absence of 90K expression was set to 100%. (C) Cell lysates were analyzed by immunoblotting. Shown is the percentage of gp120, as measured by Infrared imaging-based quantification of the amount of gp120 per (gp120 + gp160). Bar diagrams show the arithmetic means ± S.E.M. of 3-4 independent experiments. The Western Blot shown is representative for one of the experiments included in the calculation. **: p < 0.02; n.s. : > 0.05 (Student’s T-Test). Click here for file
