# Supplementary figures and images for: 90K, an interferon-stimulated gene product, reduces the infectivity of HIV-1
Source: Retrovirology. 2013 Oct 24;10:111. doi: 10.1186/1742-4690-10-111 (PMC3827937; doi:10.1186/1742-4690-10-111)

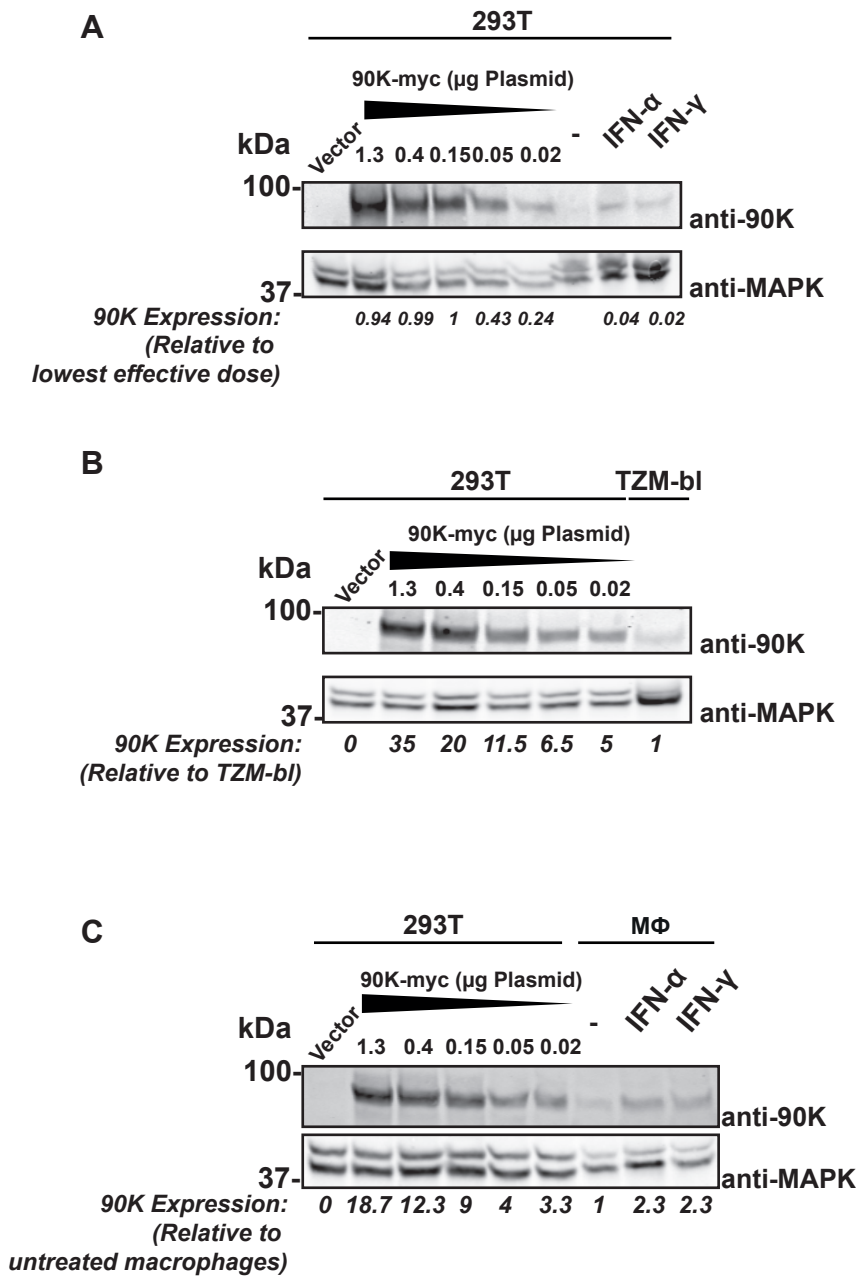

Supplement: Additional file 1: Figure S1 — Heterologous 90K levels compared to endogenous 90K levels. (A) 293T cells were cotransfected with various amounts of pcDNA6.90K-myc (1.3, 0.4, 0.15, 0.05, 0.02 μg), stimulated with 100 Units IFN-α, IFN-γ for 48 h, or left untreated. (B) Western Blot of cell lysates of 293T cells overexpressing various amounts of 90K-myc (A) and untreated TZM-bl cells. (C) Western Blot of cell lysates of 293T cells overexpressing various amounts of 90K-myc (A) and primary macrophages stimulated with 100 IFN-α, IFN-γ for 48 h, or left untreated. The numbers depict the relative 90K expression, measured by normalization of the 90K signal to the MAPK signal obtained by Infrared-imaging based quantification. The ratio obtained for the lowest antivirally active 90K plasmid dose (0.15 μg, A), the ratio obtained for TZM-bl cell lysates (B) or the ratio obtained for untreated macrophages (C) was set to 1, respectively. [file 1742-4690-10-111-S1.pdf]

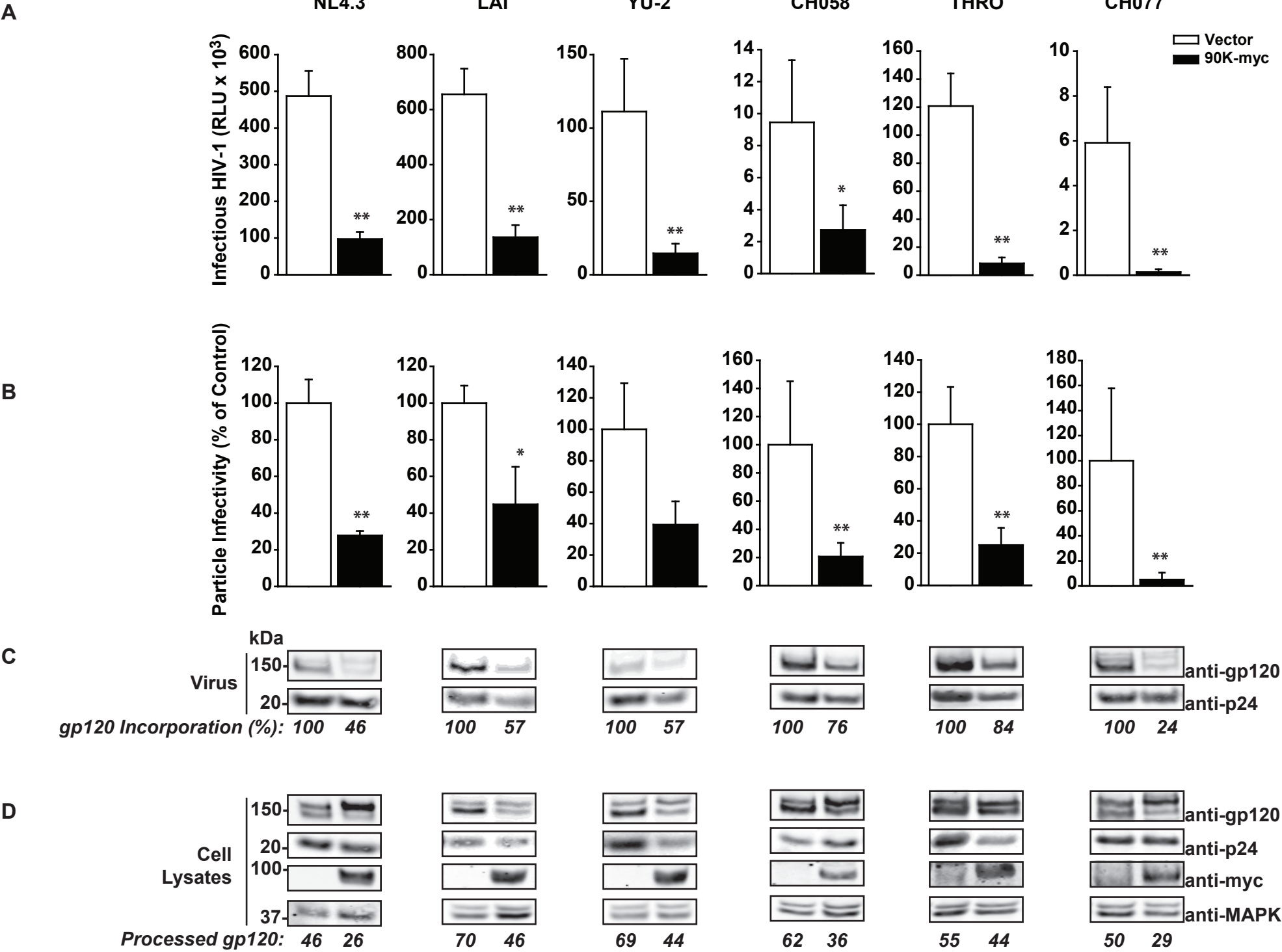

Supplement: Additional file 2: Figure S2 — 90K reduces the particle infectivity of multiple HIV-1 strains. (A) 293T cells were cotransfected with the indicated proviral plasmids and highest amount of pcDNA6.90K-myc or empty vector. Supernatants were analyzed for infectious HIV-1 using a luminometric TZM-based luciferase assay. Shown are the results of one representative experiment out of three-six. (B) Relative levels of particle infectivity, defined as HIV-1 infectivity per ng p24 capsid are depicted. (C) Sucrose cushion-purified virions were analyzed by immunoblotting. Percentages indicate the relative gp120 incorporation, as measured by Infrared imaging-based quantification of the amount of gp120 per p24. The signal intensity in absence of 90K expression was set to 100%. (D) Cell lysates were analyzed by immunoblotting using the indicated antibodies. Numbers indicate the efficiency of gp160 processing. * : p < 0.05; **: p < 0.02 (Student’s T-Test). [file 1742-4690-10-111-S2.pdf]

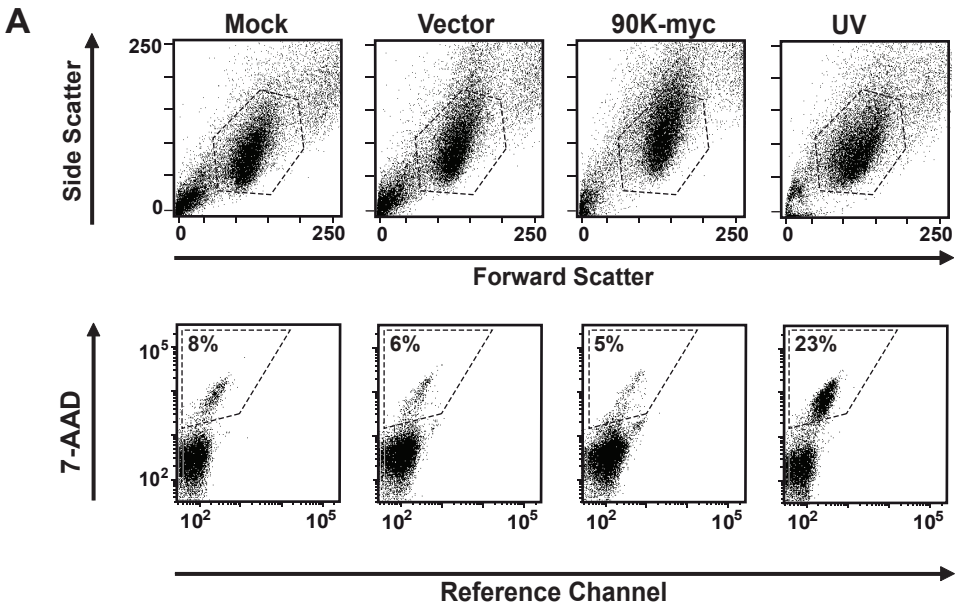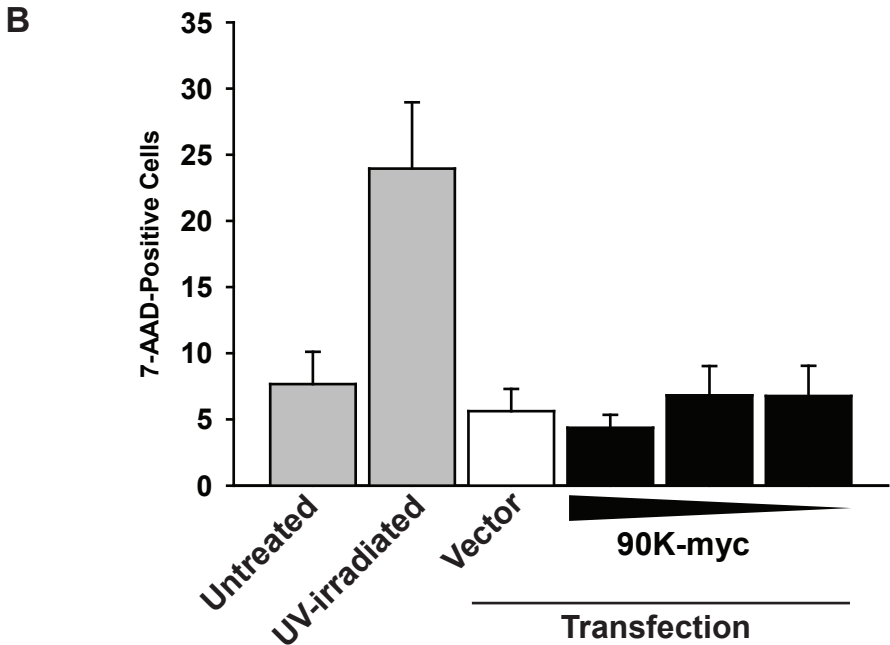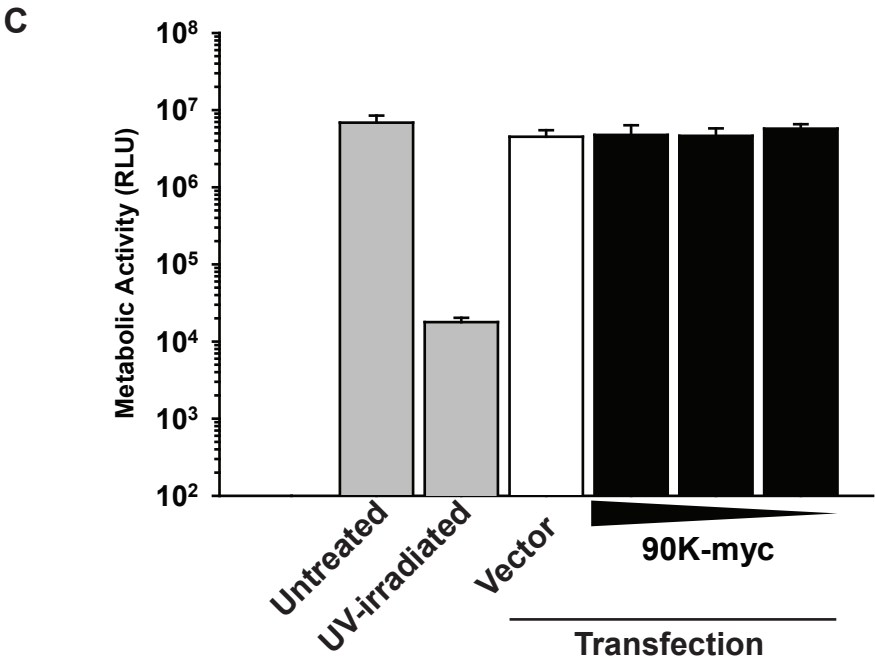

Supplement: Additional file 3: Figure S3 — 90K-myc expression is not associated with toxicity or reduced metabolic activity. (A) 293T cells were transfected with pcDNA6.90K-myc (1.3 μg), empty vector, or UV-irradiated and stained two days post transfection and one day post UV-irradiation with 7-AAD. Shown are representative dot plots of one experiment out of two. Numbers indicate percentage of 7-AAD-positive cells. (B) Quantification of 7-AAD FACS analysis. (C) Cells were lysed and analysed for metabolic activity by Cell Titer Glow assay. Shown are the RLU of triplicates obtained from one representative experiment out of two. [file 1742-4690-10-111-S3.pdf]

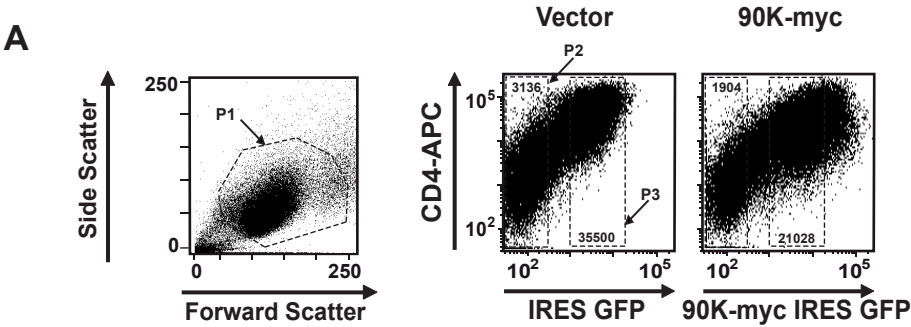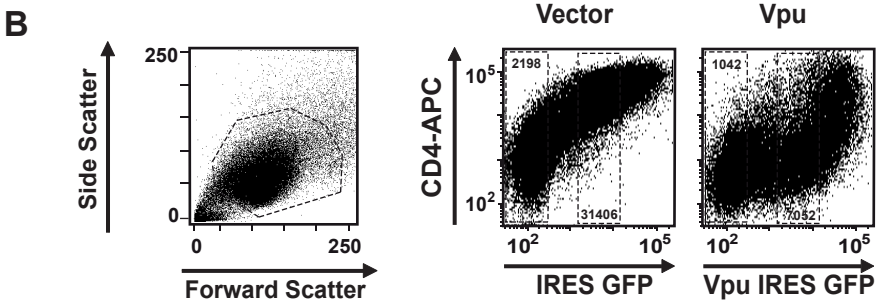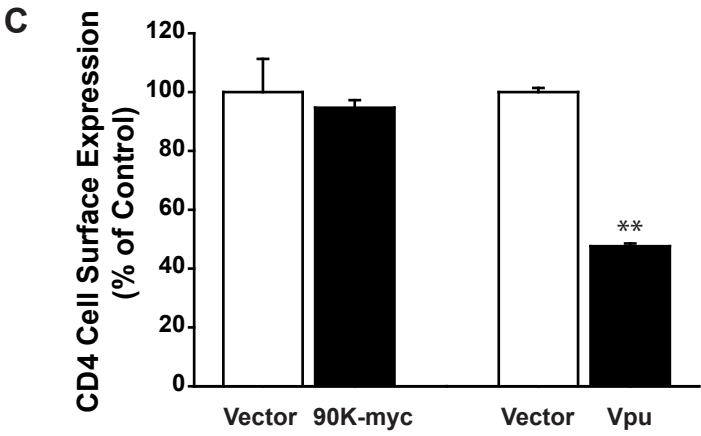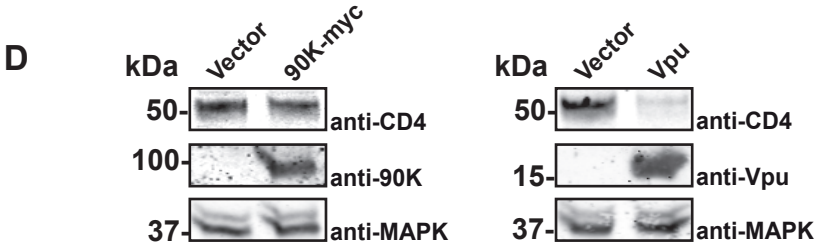

Supplement: Additional file 4: Figure S4 — 90K does not reduce the cell surface levels of CD4. (A) 293T cells were cotransfected with pcDNA.CD4 and pIRES2EGFP.90K-myc or empty vector, Cells were stained with APC-conjugated anti-CD4 and analyzed by flow cytometry. Shown are representative dot plots of one experiment out of three. (B) 293T cells were cotransfected with pcDNA.CD4 and pVpu-IRES GFP or empty vector and processed like in (A). (C) CD4 cell surface levels were calculated by comparing, within the same sample, CD4 levels on non-GFP-expressing cells (gate P2) with CD4 levels on cells with medium-high GFP expression levels (gate P3). CD4 levels on vector transfected cells were set to 100%. (D) An aliquot of the cells shown in (A) and (B) were lyzed and analyzed by Western Blotting using the indicated antibodies. [file 1742-4690-10-111-S4.pdf]

**A**

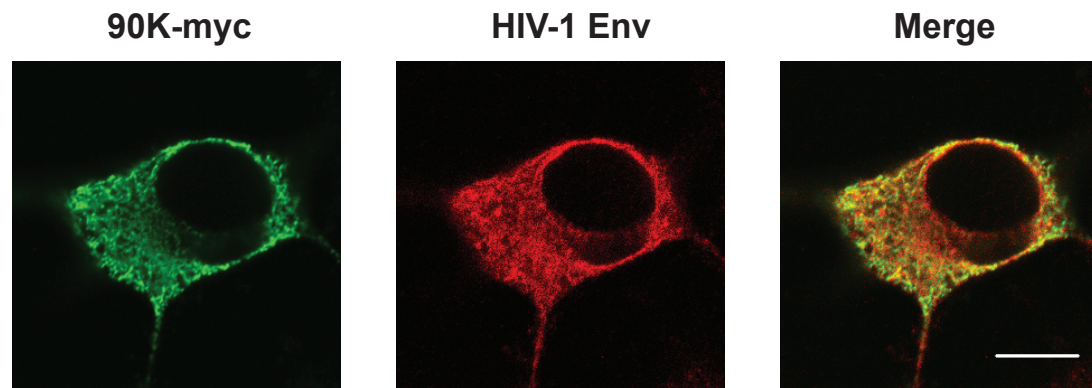

**B**

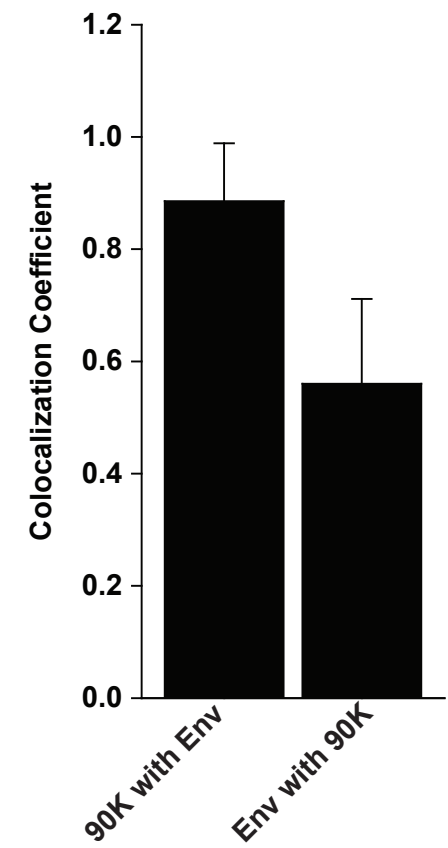

Supplement: Additional file 5: Figure S5 — 90K and Env colocalize to a high extent. (A) 293T cells were cotransfected with pcDNA6.90K-myc and an HIV-1 Env expression plasmid, and stained for 90K-myc (green) and Env (red). Scale bar: 10 μm. (B) The classic colocalization coefficient was calculated for the colocalization of 90K protein with Env protein or vice versa using ZEN2010 software. The data represent the arithmetic mean ± S.D. of 105 analyzed cells. [file 1742-4690-10-111-S5.pdf]

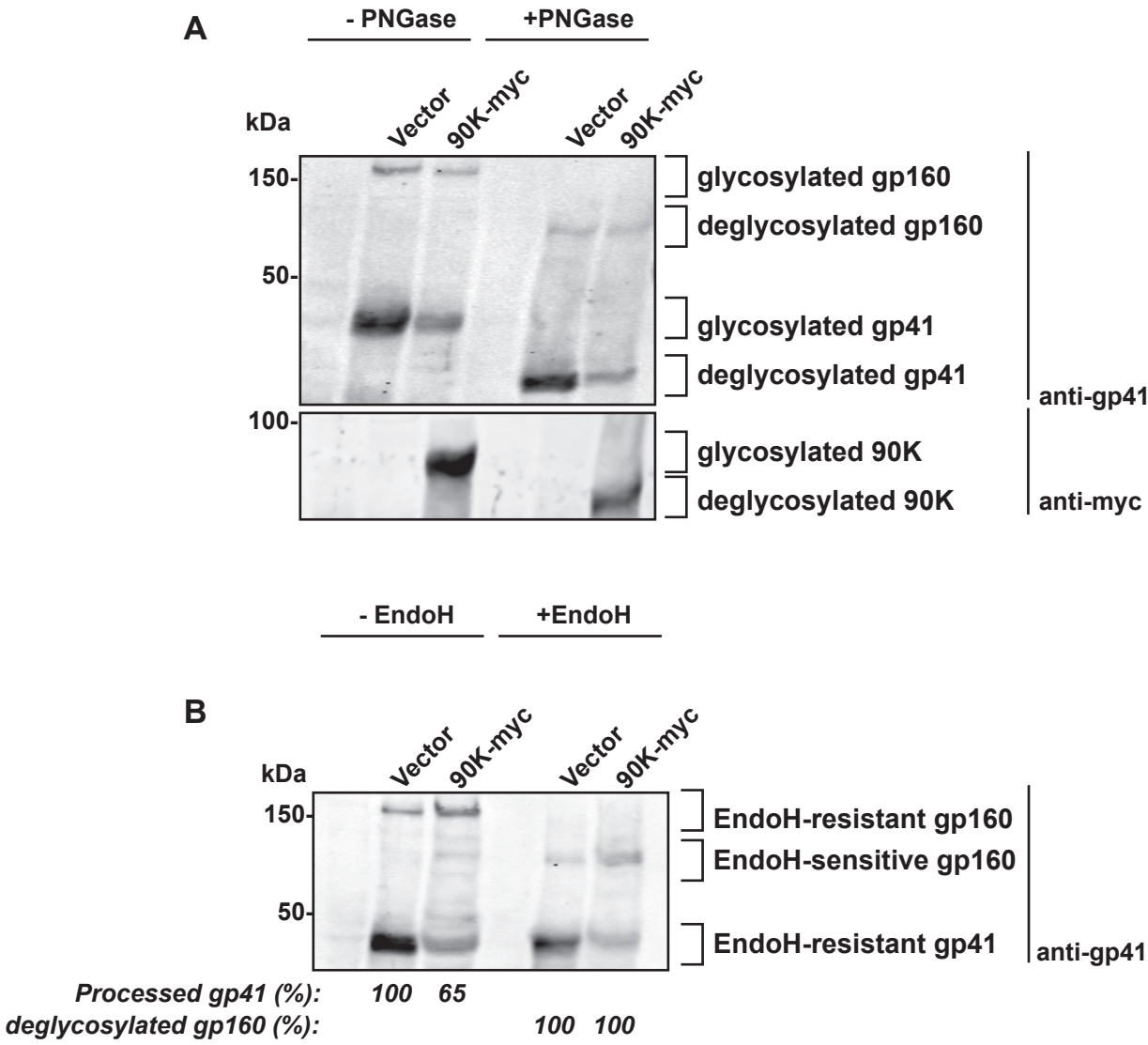

Supplement: Additional file 7: Figure S7 — 90K does not retain Env in the ER. (A-B) 293T cells were cotransfected with pBR.HIV-1 IRES GFP and vector or pcDNA6.90K-myc. (A) Cell lysates were treated with PNGase. (B) Cell lysates were treated with EndoH. Deglycosylated and control proteins were analyzed by Western Blot. Numbers indicate the efficiency of gp41 processing, calculated as the signal ratio of gp41 relative to (gp41 + gp160), or the percentage of deglycosylated gp160 to the total gp160 signal. [file 1742-4690-10-111-S7.pdf]

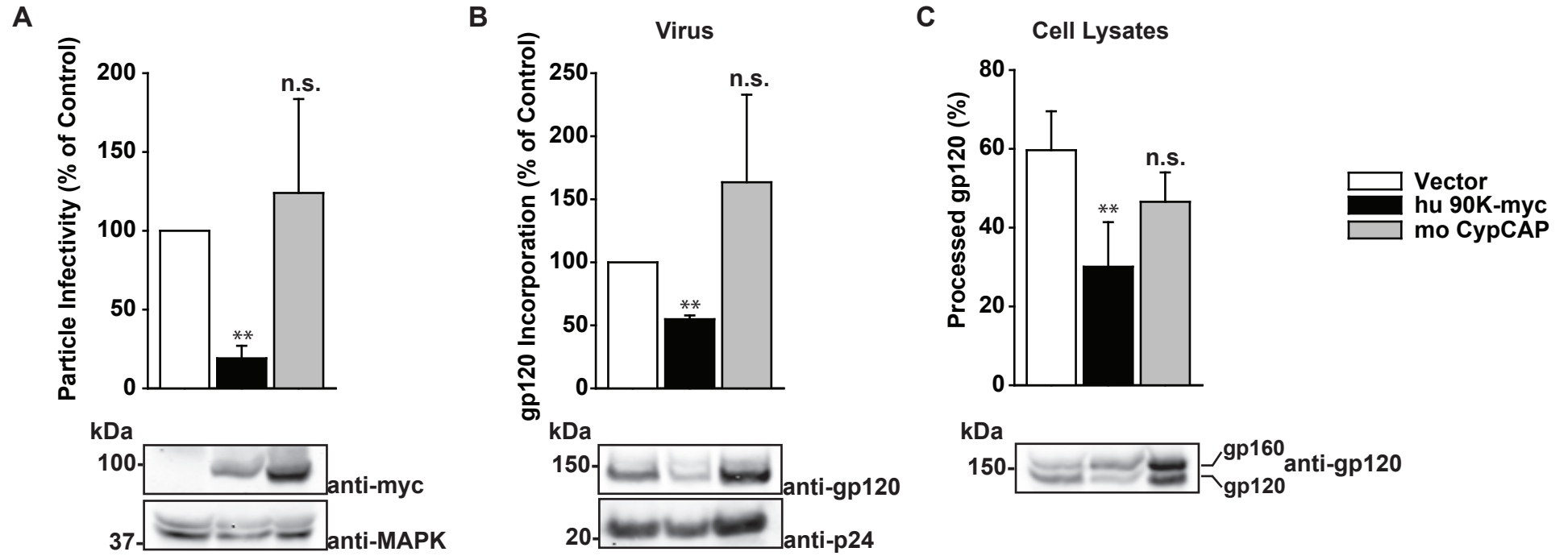

Supplement: Additional file 8: Figure S8 — Species specificity of 90K-imposed antiviral activity. (A) 293T cells were cotransfected with pBR.HIV-1 NL4.3-IRES GFP and 1.3 μg of the indicated expression constructs or empty vector. Two days post transfection, supernatants were analyzed for particle infectivity, defined as infectivity per ng p24. Cell lysates were analyzed by immunoblotting. (B) Sucrose cushion-purified virions were analyzed by immunoblotting. Shown is the relative gp120 incorporation, as measured by Infrared imaging-based quantification of the amount of gp120 per p24. The signal intensity in absence of 90K expression was set to 100%. (C) Cell lysates were analyzed by immunoblotting. Shown is the percentage of gp120, as measured by Infrared imaging-based quantification of the amount of gp120 per (gp120 + gp160). Bar diagrams show the arithmetic means ± S.E.M. of 3-4 independent experiments. The Western Blot shown is representative for one of the experiments included in the calculation. **: p < 0.02; n.s. : > 0.05 (Student’s T-Test). [file 1742-4690-10-111-S8.pdf]
