# Supplementary material for: 90K, an interferon-stimulated gene product, reduces the infectivity of HIV-1
Source: Retrovirology. 2013 Oct 24;10:111. doi: 10.1186/1742-4690-10-111 (PMC3827937; doi:10.1186/1742-4690-10-111)

Lodermeyer *et al.*, Additional File 5

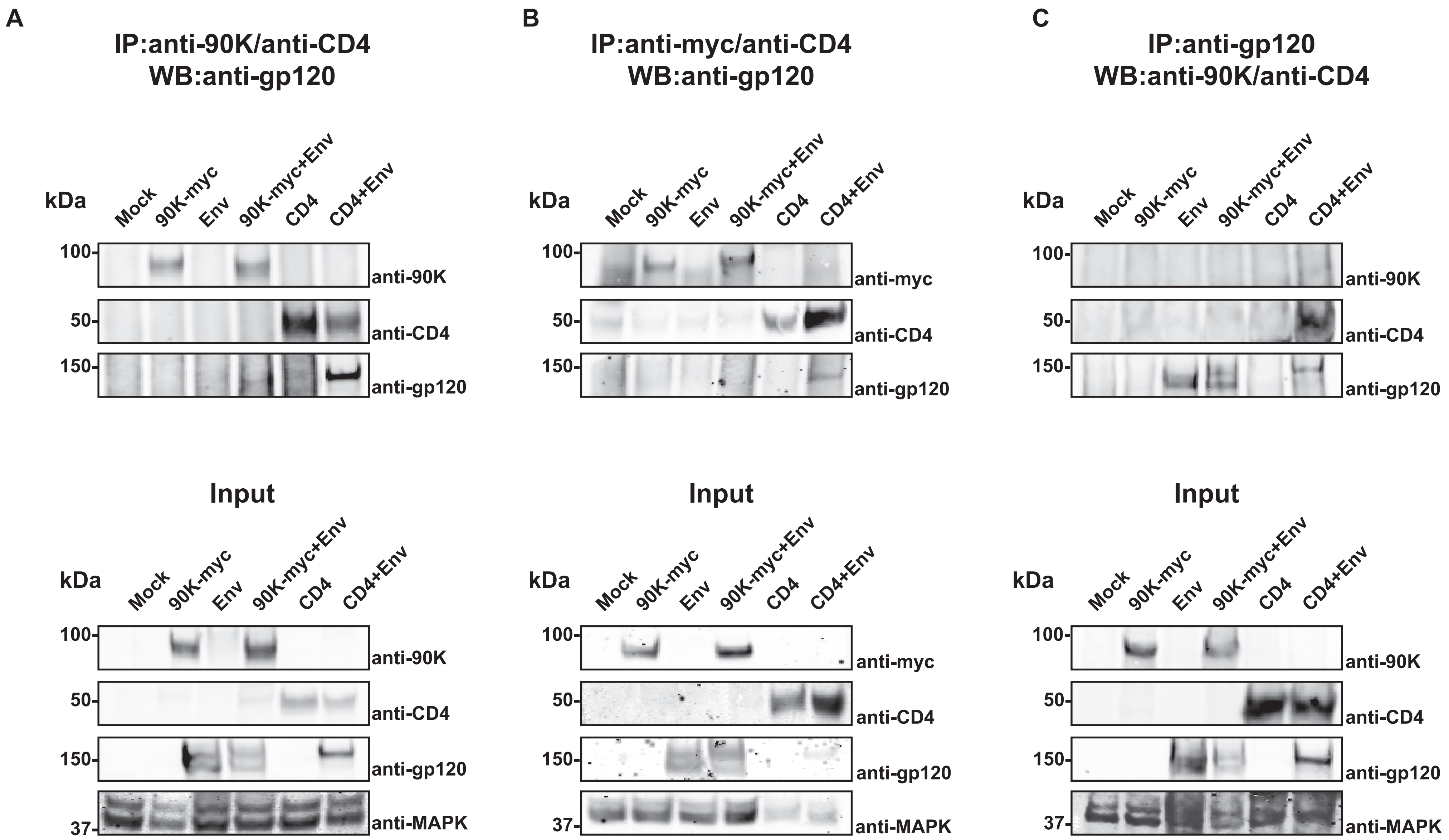

Supplement: Additional file 6: Figure S6 — No evidence for a direct interaction of 90K and HIV-1 Env. (A-C) 293T cells were cotransfected with pcDNA6, pcDNA6.90K-myc, an HIV-1 Env expression plasmid, pcDNA.CD4 or a combination out of these. (A) 90K, CD4 and bound proteins were precipitated from cell lysates by an anti-90K or anti-CD4 antibody, respectively. (B) 90K, CD4 and bound proteins were precipitated from cell lysates by an anti-myc or anti-CD4 antibody, respectively. (C) Env and bound proteins were precipitated from cell lysates by an anti-gp120 antibody. For each experimental set up, an aliquot of whole cell lysate for expression control (Input) and the precipitated proteins were analyzed by Immunoblot with indicated antibodies. [file 1742-4690-10-111-S6.pdf]
